# Degree of anisogamy is unrelated to the intensity of sexual selection

**DOI:** 10.1038/s41598-021-98616-2

**Published:** 2021-09-30

**Authors:** Judit Mokos, István Scheuring, András Liker, Robert P. Freckleton, Tamás Székely

**Affiliations:** 1 MTA-ELTE Theoretical Biology and Evolutionary Ecology Research Group, Eötvös Loránd University, Budapest, Hungary; 2 Institute of Evolution, Centre for Ecological Research, Budapest, Hungary; 3 MTA-PE Evolutionary Ecology Research Group, University of Pannonia, Veszprém, Hungary; 4 Behavioral Ecology Research Group, Center for Natural Science, University of Pannonia, Veszprém, Hungary; 5 Department of Animal and Plant Sciences, University of Sheffield, Sheffield, UK; 6 Department of Biology and Biochemistry, Milner Centre for Evolution, University of Bath, Bath, UK; 7 Department of Evolutionary Zoology and Human Biology, University of Debrecen, Debrecen, Hungary

**Keywords:** Ecology, Evolution, Zoology

## Abstract

Males and females often display different behaviours and, in the context of reproduction, these behaviours are labelled sex roles. The Darwin–Bateman paradigm argues that the root of these differences is anisogamy (i.e., differences in size and/or function of gametes between the sexes) that leads to biased sexual selection, and sex differences in parental care and body size. This evolutionary cascade, however, is contentious since some of the underpinning assumptions have been questioned. Here we investigate the relationships between anisogamy, sexual size dimorphism, sex difference in parental care and intensity of sexual selection using phylogenetic comparative analyses of 64 species from a wide range of animal taxa. The results question the first step of the Darwin–Bateman paradigm, as the extent of anisogamy does not appear to predict the intensity of sexual selection. The only significant predictor of sexual selection is the relative inputs of males and females into the care of offspring. We propose that ecological factors, life-history and demography have more substantial impacts on contemporary sex roles than the differences of gametic investments between the sexes.

## Introduction

Behavioural and physiological differences between males and females in reproduction are common, as males and females often display different mate acquisition tactics, pair-bonding strategies and offspring care^1–3^. These different strategies, termed sex roles^4,5^, encapsulate behaviours associated with intrasexual competition, intersexual mate choice, pair-bonding and parenting^6,7^. There is an immense variation in aspects of sex roles across taxa and, although exceptions to the common patterns are abundant^8,9^, in most species males tend to compete more intensively for mates than do females whereas it is typically the female who provides care for the young^10–13^.

**Figure 1:**
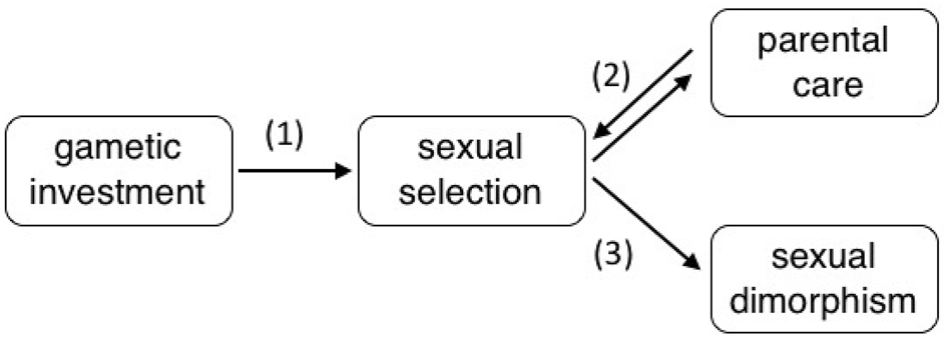
Schematic illustration of the Darwin–Bateman paradigm based on Janicke et al.^23^. The bias in gametic investment between males and females is assumed to lead to different reproductive rates of males and females, and hence to intense sexual selection among males (pathway 1), female-biased parenting (pathway 2), and elaborated trait expression in males (pathway 3). The figure was created using Keynote https://www.apple.com/uk/keynote/.

Debates about the evolutional origin of sex roles dates back to Darwin, who suggested sex differences originated in differences in gametes, as male gametes tend to be small and motile, while female gametes are usually larger and sessile^8^. He argued that the difference in gametes led to a difference in the strength of sexual selection. Working with *Drosophila,* Bateman showed that males have a higher variance in reproductive success than females, therefore males experience stronger sexual selection^14^ (however, later this example has been questioned both in term of statistics^15^ and biology^16^, although theoretically, under certain conditions it seems to be true^17^). Bateman, building on Darwin’s framework, explained this phenomenon with the different gametic investments, as females’ reproductive success is limited by the number of costly eggs produced, while for males the number of mates is a limiting factor rather than the relatively cheap sperm production. Dewsbury proposed to merge these ideas into a so-called Darwin–Bateman paradigm^18^. In sum, the Darwin–Bateman paradigm argues that anisogamy causes stronger sexual selection in males that leads to female-biased parental care and male-biased sexual dimorphism (Fig. 1)^14,18–22^.

Although some aspects of the Darwin–Bateman paradigm are well investigated, studies often produced somehow inconsistent results^18–22^ and according to our knowledge, the whole Darwin–Bateman paradigm has not been studied using life history trait data. Furthermore, both the elements and the directionality of the evolutionary cascade proposed by the paradigm have been debated^24–28^. For example, some studies supported the first step of the paradigm (i.e., anisogamy leads to sex roles)^3,29,30^ whereas other studies refuted this step^4,31^. The potential evolutionary paths from anisogamy to sexual selection has also been under discussion^32–34^ and empirical studies aiming to find associations between anisogamy and sexual selection have produced conflicting results^35,36^. In addition, the direction of the relationship between sexual selection and parenting behaviour (Fig. 1, pathway 2) is contested, for example, sexual selection and sexually antagonistic coevolution between males and females may lead to sex difference in parenting, rather than vice versa^19,30,37,38^. The most recent comprehensive evaluation of the Darwin–Bateman paradigm by Janicke et al.^23^ investigated two out of the three major pathways, and using meta-analytical methods showed that (1) the intensity of sexual selection is biased toward males, and (2) sexual selection is related to both parental care and sexual dimorphism (pathways 2 and 3, respectively, in Fig. 1).

Studying the evolutionary mechanism behind the Darwin–Bateman paradigm and the validity of the paradigm are challenging, especially as data are needed on a range of traits that are difficult to measure and obtain. Added to this, a problem is that not all traits are available for all species. Missing data are a common limitation of comparative analysis, so that typically analyses focus on the set of species for which all traits are measured. However, this could lead to possible bias if missing data are not random and reduced sample size may lead to reduced statistical power^39–42^. The latter is especially problematic when data fail to reject statistical hypotheses, as lack of power cannot be ruled out. Therefore, where possible, the impacts of missing data should be mitigated.

Here we first investigate the primary step of the Darwin–Bateman paradigm, the association between anisogamy and intensity of sexual selection (pathway 1 in Fig. 1), which was not studied by Janicke et al.^23^. This is a crucial assumption of the paradigm, and it has been tested previously only in a laboratory study of four *Drosophila* species^36^; therefore, the relevance of pathway 1 has remained to be tested across a broad range of species using data from wild populations. Second, we also explore the relationships between gametic investment, sexual selection, parental care and sexual dimorphism testing all three major pathways of the Darwin–Bateman paradigm (see Fig. 1), using bivariate and multi-predictor phylogenetically corrected statistical models.

## Materials and methods

### Sexual selection indices

We extracted data on the intensity of selection in 66 species from Janicke et al.^23^. An extensive review of the literature published since then revealed only one additional relevant study^43^. Hermaphrodite species were excluded, resulting in a dataset of 64 species. For the details of the literature review see the supplementary material.

Following Janicke et al.^23^ three indices were used to estimate the intensity of selection: opportunity for selection (*I*) that is the standardized variance in reproductive success; opportunity for sexual selection (*I*_*s*_) that is the standardized variance in mating success; and the Bateman gradient (*β*_*ss*_) that is the slope of an ordinary least-squares regression of reproductive success on mating success. For statistical calculations, the effect sizes of these three indices (*ΔI, ΔI*_*s*_, and *Δβ*_*ss*_) were used following Janicke et al*.* These indices represent bias in the intensity of selection between males and females, with positive values indicating more intense selection in males. *ΔI* and *ΔI*_*s*_ are the coefficients of variation ratio “lnCVR”, defined as the natural logarithm of the ratio between the coefficients of variation from males and females, and *Δβ*_*ss*_ was calculated as *Hedges’ d* as described in^44,45^*.* Except for the species that was added by us (*Lamprotornis superbus*), all selection indices were taken from Janicke et al.

### Sexual dimorphism

Sexual size dimorphism (SSD) was calculated as *log[male size / female size]*, which provides a statistically appropriate measure of dimorphism^46^. Thus, positive values indicate species where males are larger than females. Size dimorphism was calculated using body weight or body length; the latter are related to each other as weight = length^3^ * *c*, where* c* is a constant which is the same or very similar in both sexes. Therefore, we assume that male weight / female weight = (male length)^3^/(female length)^3^. If body size of males and females were available in different dimensions (for example male body length and female body weight), one body size measure was converted to the other. For 20 and 13 species we were not able to find reliable body size data for females or males, respectively, and therefore, SSD was not calculated for these species. The rationale for using male size relative to female size relies on studies that show intense sexual selection and polygamous mating is often, but not always, associated with male-biased sexual size dimorphism^47–51^. Also, recent studies show that sexual size dimorphism predicts sexual selection in a wide range of taxa^52,53^. Sexual size dimorphism is thus an objective and accessible measurement of the extent of sexual dimorphism and it is often used as an indicator of sexual dimorphism^53^. Furthermore, we kept SSD in our dataset to be consistent with the previous analyses of sexual selection by Janicke et al.^23^.

### Parental care bias

A five-point-scale was used to estimate parental care bias based on Liker et al.^35^: 0—female-only care, 1—1–33% male care, 2—34–66% male care, 3—77–99% male care, 4—male-only care. These scores were based either on quantitative data whenever these data were available or on qualitative description of parental care: see the justification in^35^. Parental care bias was scored for those species that exhibit some level care (N = 37 species), so species that exhibit no care were not included in parental care models (N = 27 species).

### Gamete size bias

Based on Liker et al.^35^, two indices were used to estimate gametic size bias between the sexes: (1) the gamete size bias index which was calculated as *log([male gamete size/male mass]/[female gamete size/female mass])*, and (2) the gametic investment bias which was calculated as log([testis size/male mass]/[female gamete size * clutch size /female mass]). Therefore, a positive value indicates a male-biased gamete size (or gametic investment), a positive value indicates a female-biased gamete size (or gametic investment). To estimate female gamete size, neonate weight or egg weight (i.e., fertilized ovum, including the weight of the shell and nutrients) were used, both in grams. For five species, egg volume was estimated from egg weight, and for one species it was estimated from dry egg mass (see supplementary material). To estimate male gamete size, sperm volume was used (for further details on calculating sperm volume see the supplementary material). For 12 species we did not find data on sperm sizes, and we used corresponding data form closely related species^54^ (see supplementary material). Clutch size (number of eggs per breeding event) and testis weight (in grams) were also collected from published studies (see supplementary material; for one species testis weight was based on testes width and length). Importantly, our dataset contains a wide range of species that include species with female-biased and with male-biased gametic investment bias, also approximately isogamous species. For more details, see supplementary material and the descriptive statistics (Figures S3 and S4).

### Phylogeny

**Figure 2:**
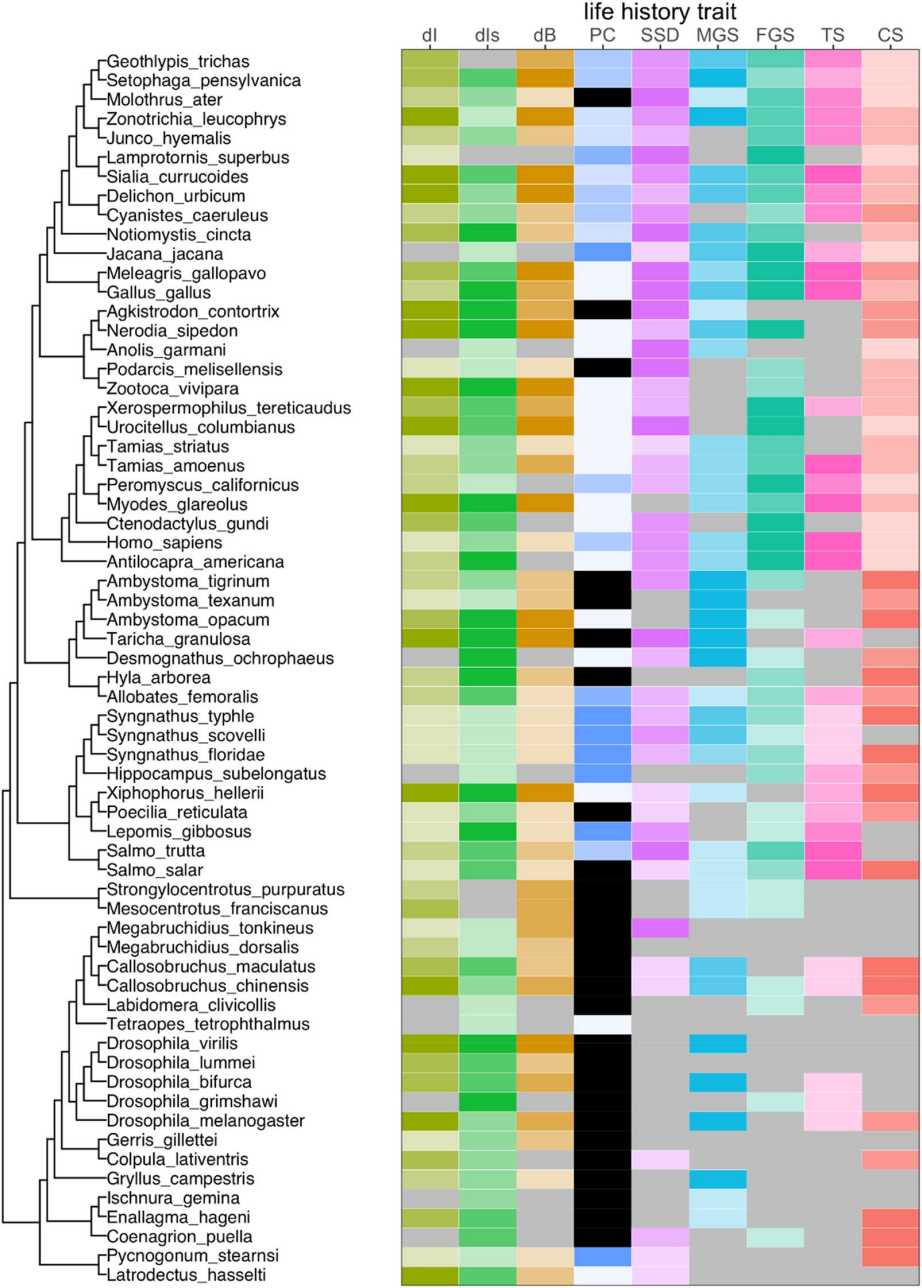
Phylogeny of species included in the study. Coloured cells indicate data extracted from Janicke et al. or the literature (see “Materials and methods”), whereas light grey cells indicats imputed data. Sexual selection was represented by three indices (dI—selection index ΔI, dIs—sexual selection index ΔI_s_, dB—Bateman gradient Δβ_ss_), and life history by six variables (PC—parental care, SSD—sexual size dimorphism, MGS—male gamete size, FGS—female gamete size, TS—testis size, CS—clutch size). For each variable, we divided the data into quartiles and each shade indicates a different quartile from low (light) to high (dark), except parental care for which five levels of parental care were indicated using different shades that range from female-only care (light blue) to male-only care (dark blue). Black indicates no care. Parental care was not imputed. The figure was prepared using RStudio version 4.0.0 https://www.R-project.org/.

To represent the phylogenetic relationships between species, the most recent comprehensive phylogeny was used from *timetree.org*^55^ that included all but seven species in the recent dataset; the latter species were added manually to the phylogeny (see Fig. 2 and supplementary material).

### Statistical methods

Phylogenetic Generalized Least Squares (PGLSs) were used to test the relationships between life history traits and sexual selection^56,57^. Based on the Darwin–Bateman paradigm, we assessed the relationships between (1) gamete size bias (or gametic investment) and sexual selection, (2) sexual selection and parental care, and (3) sexual selection and sexual size dimorphism. In addition, multi-predictor models were constructed in which one of three indices of sexual selection was the dependent variable, and gamete size bias (or gametic investment), parental care bias and sexual size dimorphism were the predictors. Pagel’s lambda was estimated using maximum likelihood to control for varying levels of phylogenetic dependence^57,58^. Note that this model accommodates a range of patterns of phylogenetic dependence, for instance, patterns that are similar to the Ornstein–Uhlenbeck (OU) model^59^. Diagnostic plots were used to check the assumptions of the models. All analyses were conducted in R using the package *caper*^60^.

### Data imputation

To use as many species for the analyses as possible, we followed previous phylogenetic analyses and imputed some of the life history trait values that were not available in the literature^41^. The following data were missing: 31% of female body mass, 20% of male body mass, 48% of testis mass, 36% of sperm length, 31% of female gamete mass, 27% of clutch size, 14% of opportunity for selection, 7% of opportunity for sexual selection and 23% of Bateman gradient (Fig. 2). In multi-predictor models, casewise deletion of species with missing data is expected to reduce the power of analysis and potentially bias the results^41^. To pre-empt these potential caveats, missing data were estimated using multiple imputation (see below) and the PGLSs were carried out using the imputed datasets as well.

Imputation is a method by which missing data is replaced with estimated data. It performs better when the proportion of missing data is low^61^. Therefore, life history trait data of additional species were collected to increase the accuracy of imputation. We carried out substantial search in the literature and found relevant data from 259 and 12,042 additional species (see Table S2 in supplementary material). Imputation was carried out using the relevant data we had for the 64 species as well as additional species (see the list of species and their data in supplementary material).

Multiple imputation was performed using a Brownian model and including Pagel’s lambda to allow for varying amounts of phylogenetic dependence. We used the *Rphylopars* R package^62,63^. The imputed means and variances were used to generate random imputed values and repeated ten times, resulting in 10 complete datasets. Each dataset was then analysed separately, and the means and standard deviations of fitted model parameters were calculated. A leave-one-out cross validation reliability check was performed to test the accuracy of the imputation by deleting the non-missing data points one by one and re-running the imputation. The correlation between the original and imputed data points was investigated (see Table 2 in supplementary material). Note that our imputation produced reliable results since the correlation coefficients are 0.8 (or above, see supplementary material).

## Results

We found no support for pathway 1 of the Darwin–Bateman paradigm (Fig. 1) since the relationships between gamete size bias, gametic investment and sexual selection are non-significant except a weak association between gamete size bias and ΔI_s_ using the original data (Table 1), although the latter association is no longer significant using the complete dataset that also includes the imputed data (Table 1). Importantly, neither gamete size bias nor gametic investment was a significant predictor of sexual selection indices in multi-predictor models (see below, Table 2). In addition, pathway 3 was also not supported since there was no relationship between sexual selection bias and sexual size dimorphism using the original dataset or the complete dataset (see Tables 1, 2).

**Table 1:** Phylogenetically corrected linear relationships (PGLSs) between components of the Darwin–Bateman paradigm using the original and the complete dataset; the latter also include imputed values.

	Original dataset				Complete dataset			
	Estimate	Standard error	t value	*p* value	Estimate	Standard error	t value	*p* value
**ΔI**	**Adjusted R**^**2**^** = 0.00, Pagel’s λ = 0.95, N = 23**				**Adjusted R**^**2**^** = −** **0.01 ± 0.01, Pagel’s λ = 0.21 ± 0.16, N = 64**			
Intercept	0.59	0.65	0.91	0.37	0.46 ± 0.03	0.15 ± 0.04	3.41 ± 1.15	0.01 ± 0.01
Gamete size bias	**−** 0.00	0.02	**−** 0.11	0.91	0.00 ± 0.01	0.01 ± 0.00	0.25 ± 0.56	0.64 ± 0.24
**ΔI**	**Adjusted R**^**2**^** = 0.03, Pagel’s λ = 0.64, N = 21**				**Adjusted R**^**2**^** = 0.01 ± 0.01, Pagel’s λ = 0.19 ± 0.17, N = 64**			
Intercept	0.60	0.31	1.93	0.07	0.45 ± 0.03	0.13 ± 0.05	4.51 ± 2.42	0.01 ± 0.01
Gametic investment bias	0.03	0.05	0.75	0.46	0.00 ± 0.01	0.01 ± 0.00	0.61 ± 1.05	0.36 ± 0.27
**ΔI**_**s**_	**Adjusted R**^**2**^** = 0.17, Pagel’s λ = 0.00, N = 24**				**Adjusted R**^**2**^** = 0.00 ± 0.01, Pagel’s λ = 0.00 ± 0.00, N = 64**			
Intercept	**−** 0.36	0.31	**−** 1.15	0.26	0.20 ± 0.03	0.09 ± 0.01	2.27 ± 0.40	0.04 ± 0.03
Gamete size bias	**−** 0.04	0.02	**−** 2.10	0.05	**−** 0.01 ± 0.00	0.01 ± 0.00	**−** 1.00 ± 0.44	0.36 ± 0.21
**ΔI**_**s**_	**Adjusted R**^**2**^** = 0.03, Pagel’s λ = 0.00, N = 21**				**Adjusted R**^**2**^** = −** **0.01 ± 0.01, Pagel’s λ = 0.00 ± 0.00, N = 64**			
Intercept	0.41	0.28	1.47	0.16	0.27 ± 0.02	0.06 ± 0.00	4.13 ± 0.25	0.00 ± 0.00
Gametic investment bias	0.04	0.06	0.70	0.49	0.00 ± 0.01	0.01 ± 0.00	**−** 0.00 ± 0.69	0.61 ± 0.26
**Δβ**_**ss**_	**Adjusted R**^**2**^** = 0.00, Pagel’s λ = 0.60, N = 22**				**Adjusted R**^**2**^** = −** **0.01 ± 0.01, Pagel’s λ = 0.10 ± 0.14, N = 64**			
Intercept	0.38	0.39	0.99	0.33	0.43 ± 0.03	0.10 ± 0.04	4.62 ± 1.52	0.01 ± 0.02
Gamete size bias	0.00	0.02	0.19	0.85	0.00 ± 0.00	0.01 ± 0.00	0.56 ± 0.58	0.60 ± 0.31
**Δβ**_**ss**_	**Adjusted R**^**2**^** = 0.01, Pagel’s λ = 0.52, N = 19**				**Adjusted R**^**2**^** = 0.0 ± 0.02, Pagel’s λ = 0.07 ± 0.12, N = 64**			
Intercept	0.31	0.29	1.08	0.30	0.41 ± 0.03	0.08 ± 0.04	6.21 ± 2.28	0.00 ± 0.01
Gametic investment bias	0.02	0.05	0.33	0.75	0.01 ± 0.01	0.01 ± 0.00	0.77 ± 0.76	0.39 ± 0.23
**Parental care bias**	**Adjusted R**^**2**^** = 0.29, Pagel’s λ = 0.99, N = 32**				**Adjusted R**^**2**^** = 0.24 ± 0.05, Pagel’s λ = 0.94 ± 0.15, N = 37**			
Intercept	2.25	1.56	1.44	0.16	1.89 ± 0.15	1.42 ± 0.28	1.46 ± 0.73	0.21 ± 0.08
ΔI	**−** 0.96	0.28	**−** 3.49	*0.00*	**−** 1.01 ± 0.19	0.29 ± 0.02	**−** 3.49 ± 0.46	*0.00* ± *0.00*
**Parental care bias**	**Adjusted R**^**2**^** = 0.20, Pagel’s λ = 0.97, N = 35**				**Adjusted R**^**2**^** = 0.19 ± 0.03, Pagel’s λ = 0.97 ± 0.02, N = 37**			
Intercept	1.59	1.37	1.16	0.26	1.60 ± 0.01	1.41 ± 0.08	1.13 ± 0.06	0.27 ± 0.02
ΔI_s_	**−** 0.89	0.31	**−** 2.89	*0.01*	**−** 0.91 ± 0.05	0.30 ± 0.01	**−** 3.04 ± 0.28	*0.01* ± *0.00*
**Parental care bias**	**Adjusted R**^**2**^** = 0.06, Pagel’s λ = 1.00, N = 29**				**Adjusted R**^**2**^** = 0.22 ± 0.05, Pagel’s λ = 0.85 ± 0.12, N = 37**			
Intercept	1.81	1.57	1.16	0.26	1.79 ± 0.20	1.10 ± 0.28	1.77 ± 0.61	0.13 ± 0.12
Δβ_ss_	**−** 0.44	0.33	**−** 1.36	0.19	**−** 1.34 ± 0.26	0.41 ± 0.04	**−** 3.31 ± 0.46	*0.00* ± *0.01*
**Sexual size dimorphism**	**Adjusted R**^**2**^** = 0.01, Pagel’s λ = 0.83, N = 44**				**Adjusted R**^**2**^** = −** **0.03 ± 0.00, Pagel’s λ = 0.99 ± 0.00, N = 64**			
Intercept	**−** 0.26	0.23	**−** 1.17	0.25	0.01 ± 0.74	0.45 ± 0.22	**−** 0.38 ± 1.37	0.45 ± 0.34
ΔI	**−** 0.04	0.08	**−** 0.48	0.63	**−** 0.01 ± 0.01	0.08 ± 0.04	**−** 0.07 ± 0.13	0.91 ± 0.07
**Sexual size dimorphism**	**Adjusted R**^**2**^** = 0.00, Pagel’s λ = 0.84, N = 44**				**Adjusted R**^**2**^** = −** **0.03 ± 0.00, Pagel’s λ = 0.99 ± 0.00, N = 64**			
Intercept	**−** 0.28	0.24	**−** 1.13	0.26	0.01 ± 0.74	0.45 ± 0.21	**−** 0.39 ± 1.38	0.45 ± 0.33
ΔI_s_	0.03	0.08	0.32	0.75	**−** 0.00 ± 0.01	0.08 ± 0.04	**−** 0.01 ± 0.09	0.95 ± 0.04
**Sexual size dimorphism**	**Adjusted R**^**2**^** = 0.01, Pagel’s λ = 0.85, N = 39**				**Adjusted R**^**2**^** = −** **0.02 ± 0.01, Pagel’s λ = 0.99 ± 0.00, N = 64**			
Intercept	**−** 0.33	0.25	**−** 1.33	0.19	**−** 0.00 ± 0.73	0.45 ± 0.21	**−** 0.41 ± 1.37	0.43 ± 0.32
Δβ_ss_	0.05	0.10	0.50	0.62	0.04 ± 0.07	0.10 ± 0.05	0.36 ± 0.56	0.63 ± 0.27

PGLS discard any species that has a missing value, therefore the sample size of the models using the original and complete dataset may differ. Parental care bias was calculated only for those species that exhibit some level of care (37 species). N refers to the number of species. For the complete dataset multiple imputation using lambda model was used to estimate missing data, 10 completed datasets were generated and PGLSs were carried out in each dataset (see “Materials and methods”). The mean and the standard deviation of the statistics are shown. For each model the response variables are in bold and the predictor variables are listed below. ΔI, ΔI_s_ and Δβ_ss_ refer to the opportunity for selection, the opportunity for sexual selection and the Bateman gradient, respectively. Statistically significant associations are in italics.

**Table 2:** Phylogenetically corrected multiple predictor relationships (PGLSs) between the bias of sexual selection and the bias of parental care, gametic investment and sexual size dimorphism, using the original and the completed datasets.

	Original dataset				Complete dataset			
	Estimate	Standard error	t value	*p* value	Estimate	Standard error	t value	*p* value
**Model 1**	**Adjusted R**^**2**^** = 0.11, Pagel’s λ = 0.94, N = 19**				**Adjusted R**^**2**^** = 0.37 ± 0.07, Pagel’s λ = 0.00 ± 0.00**			
Intercept	0.48	0.80	0.60	0.56	0.78 ± 0.12	0.22 ± 0.03	3.57 ± 0.89	0.01 ± 0.03
Gamete size bias	**−** 0.02	0.04	**−** 0.45	0.66	0.00 ± 0.01	0.01 ± 0.00	0.02 ± 0.47	0.77 ± 0.22
Sexual size dimorphism	**−** 0.66	0.97	**−** 0.68	0.51	**−** 0.13 ± 0.13	0.15 ± 0.06	**−** 0.81 ± 0.72	0.43 ± 0.28
Parental care bias	**−** 0.15	0.12	**−** 1.18	0.26	**−** 0.23 ± 0.03	0.05 ± 0.00	**−** 4.67 ± 0.79	*0.00* ± *0.00*
**Model 2**	**Adjusted R**^**2**^** = 0.44, Pagel’s λ = 0.00, N = 17**				**Adjusted R**^**2**^** = 0.38 ± 0.07, Pagel’s λ = 0.00 ± 0.00**			
Intercept	1.10	0.31	3.59	0.00	0.76 ± 0.06	0.11 ± 0.01	6.73 ± 0.96	0.00 ± 0.00
Gamete size bias	0.10	0.07	1.52	0.15	**−** 0.00 ± 0.01	0.01 ± 0.00	**−** 0.09 ± 0.99	0.48 ± 0.24
Sexual size dimorphism	**−** 0.93	1.28	**−** 0.72	0.48	**−** 0.08 ± 0.12	0.15 ± 0.05	**−** 0.47 ± 0.64	0.67 ± 0.33
Parental care bias	**−** 0.18	0.08	**−** 2.29	*0.04*	**−** 0.23 ± 0.03	0.05 ± 0.00	**−** 4.70 ± 0.86	*0.00* ± *0.00*
**Model 3**	**Adjusted R**^**2**^** = 0.54, Pagel’s λ = 0.00, N = 20**				**Adjusted R**^**2**^** = 0.27 ± 0.04, Pagel’s λ = 0.00 ± 0.00**			
Intercept	0.15	0.44	0.35	0.73	0.45 ± 0.18	0.24 ± 0.04	1.91 ± 0.82	0.15 ± 0.25
Gamete size bias	**−** 0.04	0.03	**−** 1.39	0.18	**−** 0.01 ± 0.01	0.01 ± 0.00	**−** 0.59 ± 0.90	0.55 ± 0.33
Sexual size dimorphism	**−** 0.13	0.62	**−** 0.20	0.84	0.02 ± 0.11	0.16 ± 0.07	**−** 0.11 ± 0.79	0.56 ± 0.25
Parental care bias	**−** 0.26	0.08	**−** 3.36	*0.00*	**−** 0.21 ± 0.01	0.05 ± 0.00	**−** 3.84 ± 0.29	*0.00* ± *0.00*
**Model 4**	**Adjusted R**^**2**^** = 0.40, Pagel’s λ = 0.00, N = 17**				**Adjusted R**^**2**^** = 0.27 ± 0.02, Pagel’s λ = 0.00 ± 0.00**			
Intercept	0.67	0.42	1.57	0.14	0.56 ± 0.07	0.12 ± 0.01	4.61 ± 0.61	0.00 ± 0.00
Gamete size bias	0.00	0.09	0.04	0.97	**−** 0.00 ± 0.01	0.01 ± 0.00	**−** 0.38 ± 1.00	0.41 ± 0.24
Sexual size dimorphism	**−** 0.24	1.84	**−** 0.13	0.90	**−** 0.01 ± 0.15	0.17 ± 0.06	**−** 0.17 ± 0.93	0.44 ± 0.23
Parental care bias	**−** 0.25	0.11	**−** 2.26	*0.04*	**−** 0.21 ± 0.01	0.05 ± 0.00	**−** 3.92 ± 0.16	*0.00* ± *0.00*
**Model 5**	**Adjusted R**^**2**^** = 0.19, Pagel’s λ = 0.44, N = 18**				**Adjusted R**^**2**^** = 0.34 ± 0.04, Pagel’s λ = 0.00 ± 0.00**			
Intercept	0.81	0.47	1.74	0.10	0.74 ± 0.14	0.19 ± 0.03	3.96 ± 0.67	0.00 ± 0.00
Gamete size bias	0.01	0.03	0.37	0.72	0.00 ± 0.01	0.01 ± 0.00	0.17 ± 0.84	0.53 ± 0.29
Sexual size dimorphism	0.05	0.70	0.07	0.94	0.06 ± 0.16	0.13 ± 0.06	0.26 ± 0.99	0.47 ± 0.28
Parental care bias	**−** 0.17	0.09	**−** 1.81	0.09	**−** 0.19 ± 0.02	0.04 ± 0.00	**−** 4.42 ± 0.45	*0.00* ± *0.00*
**Model 6**	**Adjusted R**^**2**^** = 0.27, Pagel’s λ = 0.35, N = 15**				**Adjusted R**^**2**^** = 0.35 ± 0.06, Pagel’s λ = 0.00 ± 0.00**			
Intercept	0.55	0.38	1.43	0.18	0.70 ± 0.06	0.10 ± 0.01	7.29 ± 0.86	0.00 ± 0.00
Gamete size bias	**−** 0.01	0.07	**−** 0.11	0.91	0.00 ± 0.01	0.01 ± 0.00	**−** 0.05 ± 1.07	0.48 ± 0.31
Sexual size dimorphism	**−** 0.41	1.30	**−** 0.32	0.76	0.08 ± 0.19	0.13 ± 0.05	0.39 ± 1.23	0.42 ± 0.26
Parental care bias	**−** 0.17	0.09	**−** 1.84	0.09	**−** 0.18 ± 0.01	0.04 ± 0.00	**−** 4.41 ± 0.47	*0.00* ± *0.00*

For each model the response variables are in bold and the predictor variables are listed below. ΔI, ΔI_s_ and Δβ_ss_ refer to the opportunity for selection, the opportunity for sexual selection and the Bateman gradient, respectively. The response variable is ΔI (Models 1 & 2), ΔI_s_ (Models 3 & 4), and Δβ_ss_ (Models 5 & 6). Parental care bias was only calculated for species that exhibit some level of care, thus the models of complete dataset used 37 species. Statistically significant results are in italic. See Table 1 for further explanation.

**Figure 3:**
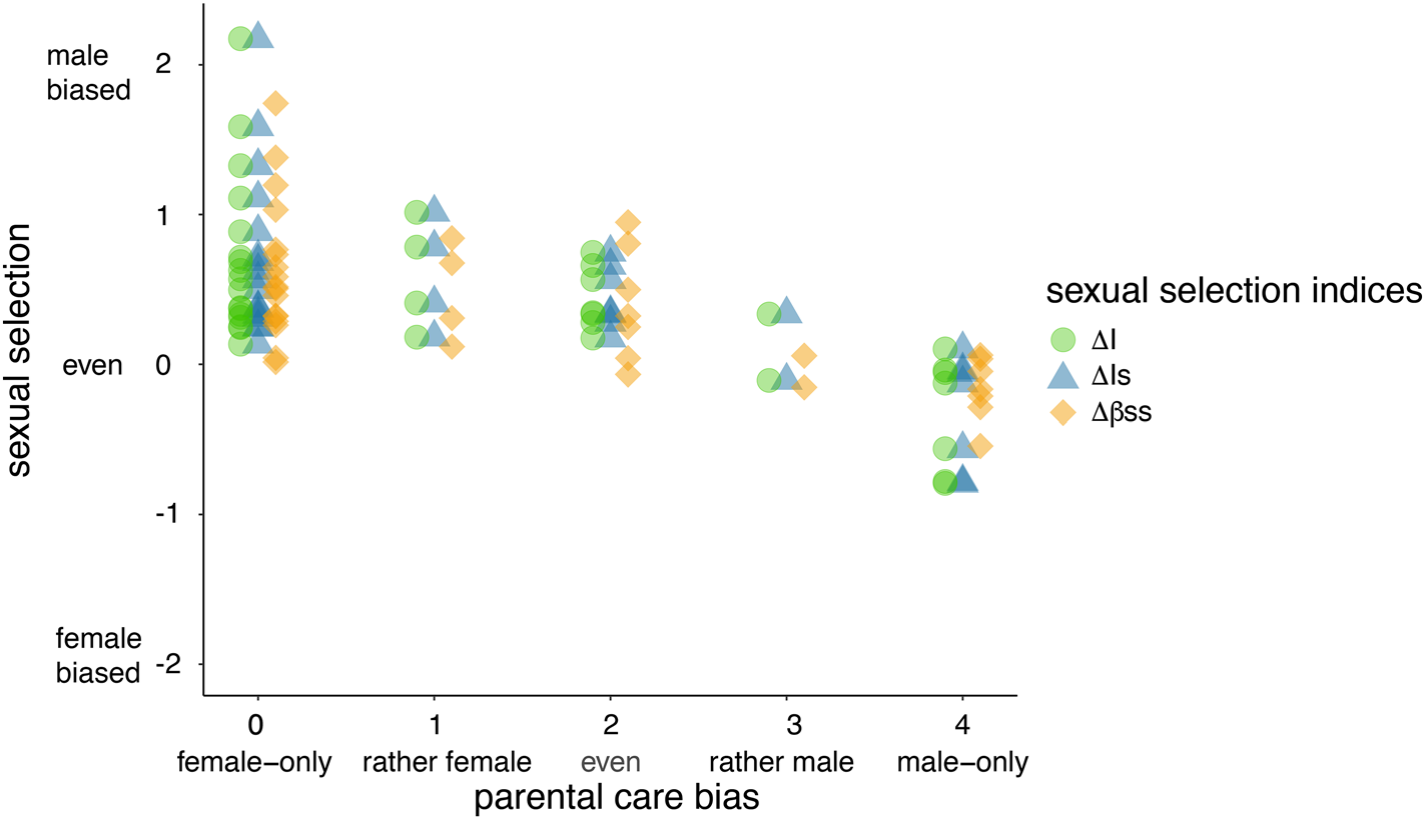
The intensity of sexual selection (*ΔI*: opportunity of selection, *ΔI*_*s*_: opportunity for sexual selection, *Δβ*_*ss*_: Bateman gradient) in relation to sex-biased parental care (N = 37 species). Stronger sexual selection on males associates with female-biased parental care; the relationship is significant for all three indices of sexual selection (see Table 1). Shaded data points represent overlapping data. No imputed data were used in the figure. The figure was prepared using RStudio version 4.0.0 https://www.R-project.org/.

However, we found consistent support for pathway 2 since parental care bias was associated with sexual selection indices (Table 1, Fig. 3). Thus if parenting shifts toward female-biased care this is associated with more intense selection on males (Fig. 3). Taken together, among the predicted relationships of the Darwin–Bateman paradigm, only pathway 2 (parental care—sexual selection) has consistent support, while pathway 1 (gametic investment—sexual selection) has weak if any support and pathway 3 (sexual dimorphism—sexual selection) is not supported.

To investigate potential co-effects among pathways, multi-predictor models were constructed in which one of three indices of sexual selection was the dependent variable, and gametic investment, parental care bias and sexual size dimorphism were the predictors. Following the results of the bivariate PGLSs, among the life history traits, only parental care bias predicted sexual selection (see Table 2). Parental care bias has a negative association with ΔI and ΔI_s_ in the original dataset, whereas in the complete dataset care bias was significantly associated with all three indices (Fig. 3). R^2^ values are moderate (0.11–0.54) suggesting that further unexplored life history and/or ecological traits may also influence the strengths of sexual selection acting on males vs females.

## Discussion

Our study provided two major results. First, we found that the degree of anisogamy does not predict the intensity of sexual selection (Table 1). Second, male-biased sexual selection is associated with female-biased parenting (Fig. 3, Table 1) supporting pathway 2 so that parental care is related to sexual selection.

The lack of relationship between gametic investment and sexual selection is consistent across three indices of sexual selection and two estimates of gametic investment (Table 1), and the results remain robust in multi-predictor models which control for the effects of other predictors (Table 2). Note that the imputed datasets that use sperm length from 489 species and testis mass from 259 species provide arguable better model estimates than the non-imputed datasets, and in all models R^2^ values are close to zero (Table 1).

We suggest three explanations for the lack of association between anisogamy and sexual selection. First, anisogamy—the existence of sexes due to different gamete sizes of males versus females that has been suggested to evolve repeatedly from isogamic ancestors^64^—may only provide the starting condition for evolving different male vs female strategies, and the relationship between sexual selection and anisogamy may be restricted to the early stages of anisogamy evolution^29^. Once anisogamy produced different sexes in dioecious organisms, then ecology, life history and demography could all impact the development of sex-specific strategies that increase male and female reproductive success^5,65^. To study these scenarios further studies are warranted that will explore the associations between ecological, life-history and demographic traits, sexual selection and gametic biases (see also^53^).

Second, anisogamy is related to sexual selection at all anisogamy ratios, although the impacts of ecology and life history possibly override the effects emerging from anisogamy. For example, the harsh environment can tone down behavioural differences between the sexes by selection for monogamy and biparental care^66^, or can enhance the difference between sexes in sexual selection^67^ although it may not directly impact male vs female gametic investments^68^. A demographic property of populations, the adult sex ratio (ASR), also impacts sex roles in birds, fish and humans since biased sex ratios induce changes in pair bonding and parenting^7,69–74^. Consistently with phylogenetic studies, recently a long-term study showed fluctuations in ASR that impacted female choice in wild populations of Darwin’s finches^75^. Experimental studies showed that female-biased ASR facilitates males to develop traits improving their competitiveness for accessing mate, and even the presence or absence of the female could trigger trait development^76^. Operational sex ratio (OSR; the ratio of sexually active males to females) may also have a pronounced effect on sex roles^77^, although OSR has been proposed to emerge from sex roles, rather than vice versa^4,6,66^.

Third, the estimates of gametic investment may not be perfect, making it difficult to detect relationships between anisogamy and sexual selection. Although comparing the energy contents of female vs male gametes would be ideal, these data do not yet exist across a broad range of taxa. A potential drawback of using gamete mass as an indicator of gametic investment is that the quality of ejaculate and female gamete (and therefore their size) may change with age and health conditions^78–82^. Furthermore, testis size varies over the breeding season^83,84^ and clutch size may respond to environmental factors^35,85–87^; so that these variations could potentially mask the predicted associations between gametic bias and sexual selection.

Our second major result is that our analyses supported only one element of the Darwin–Bateman paradigm, that parental care is related to sexual selection, since male-biased sexual selection is associated with female-biased parenting (Fig. 3, Table 1). Consistently in multi-predictor models, the only variable that predicts sex difference in sexual selection is parental care bias (Table 2). The significant relationship between parental care and sexual selection supports previous phylogenetic analyses^35,87–91^ and overall, it is consistent with the results of Janicke et al.^23^. We acknowledge that parental care and sexual selection may have a more complex relationship than usually thought^37,92^: parental care may impact the intensity of sexual selection as Trivers^93^ originally proposed, although sexually selected traits and mating opportunities may also drive parenting decisions^94–96^. Note that we prefer to use the term parental care as opposed to parental investment, since the latter assumes that caring is costly; an assumption that is often not tested thoroughly^97^.

The association between sexual selection and care is complex and may emerge via the ASR or the OSR. Parental care may be costly for the caring sex that leads to higher mortality, therefore sex-biased care may produce biased ASR that has a knock-on effect on intrasexual competition; however if the mortality is higher during mating competition than during care, the common sex is predicted to perform more care while the rare sex is expected to spend more time in the mating pool^4,98^. Alternatively, changes in OSR could impact sexual selection^99^: as the caring sex becomes a limited source for the other sex as a mating partner, biased care leads to biased OSR that influence the intensity of competition^100,101^. Since OSR and ASR are expected to be associated (although empirical studies do not always support this association^102^) to distinguish the impacts of ASR from OSR will require further investigations.

We propose three potential reasons for the lack of correlation between sexual size dimorphism and sexual selection. First, sexual size dimorphism could be due to various selective processes, not only sexual selection, including natural selection and fecundity selection^50,51,103^. Also, sexual selection may not have a monotonous relationship with sexual size dimorphism (as we assumed in the current study), because if sexual selection is manifested via the agility of males^104^ than intense sexual selection produces small males relative to females^105^. Furthermore, body size itself may not be under sexual selection^34,106^. Second, a possible explanation for the difference between Janicke et al.^23^ who reported an association between sexual dimorphism and sexual selection and our result, is that Janicke et al.^23^ used a composite scoring system for dimorphism that included ornamentation and behaviour, whereas we used only one variable, body size, to represent dimorphism (or the lack of dimorphism). It is conceivable that ornaments and behaviour (such as courtship) are more directly related to some forms of sexual selection than body size dimorphism^104,106^. Therefore, the combined effects of ornamentation and behaviour could produce an association with sexual selection whereas body size dimorphism alone may not. Third, sexual size dimorphism could be reduced in species that do not have parental care behaviour^88^. By using a continuous parental care bias variable, this may have led to restricting the analyses to species that exhibit care. As species that do not have parenting behaviour play a major role to create a correlation between parental care and sexual dimorphism, excluding them may have reduced the ability to spot the association between dimorphism and sexual selection.

Nevertheless, a limitation of our study as well as those of Janicke et al.^23^ is that the 64 species used in these studies may not fully represent the diversity of gametic traits nor sexual selection exhibited by the huge diversity of extant terrestrial and aquatic animals. Therefore, future analyses of the Darwin–Bateman paradigm are warranted by using more detailed analyses of gametic investment, sexual selection and parenting in an ecological and demographic framework.

Ideally, life-history traits from high number of taxonomically diverse species should be used to test the Darwin–Bateman paradigm. Even though we aimed at using the best available data, we had numerous missing data therefore imputation was used instead of removing species with missing data to keep as many species in the analyses. Imputation becomes a frequently used tool in ecology and evolutionary biology as an alternative to removing species with missing observations^107–113^. The use and reliability of imputation in ecology and evolutionary biology are discussed in several articles^40,45,61,114,115^, and it is suggested as a reliable method for estimating missing data in ecological datasets^116,117^. As the analyses of the original and the imputed dataset gave similar results nearly all cases, we argue our results are reliable.

In conclusion, our study confirms and extends the findings of Janicke et al.^23^ by showing parental care bias predicts sexual selection. However, the results do not support a key assumption of the Darwin–Bateman paradigm since the extent of anisogamy is unrelated to the intensity of sexual selection. We argue that ecological, life history and demographic variables could influence sex roles, and we call for new studies that integrate these processes into the investigation of sex role evolution.

## Supplementary Information

Supplementary Information.

## Data Availability

The codes are available at https://datadryad.org/stash/dataset/doi:10.5061%2Fdryad.fqz612jpp.
