# Author Correction: Degree of anisogamy is unrelated to the intensity of sexual selection

**DOI:** 10.1038/s41598-021-02778-y

**Published:** 2021-11-30

**Authors:** Judit Mokos, István Scheuring, András Liker, Robert P. Freckleton, Tamás Székely

**Affiliations:** 1 MTA‑ELTE Theoretical Biology and Evolutionary Ecology Research Group, Eötvös Loránd University, Budapest, Hungary; 2 Institute of Evolution, Centre for Ecological Research, Budapest, Hungary; 3 MTA‑PE Evolutionary Ecology Research Group, University of Pannonia, Veszprém, Hungary; 4 Behavioral Ecology Research Group, Center for Natural Science, University of Pannonia, Veszprém, Hungary; 5 Department of Animal and Plant Sciences, University of Sheffield, Sheffield, UK; 6 Department of Biology and Biochemistry, Milner Centre for Evolution, University of Bath, Bath, UK; 7 Department of Evolutionary Zoology and Human Biology, University of Debrecen, Debrecen, Hungary

Correction to: *Scientific Reports* https://doi.org/10.1038/s41598-021-98616-2, published online 30 September 2021

The original version of this Article contained an error in the Code availability section, where.

“The dataset analysed during the current study are available from the corresponding author on reasonable request and will be placed to a public repository and/or be downloadable as a supplementary file and be available from the date of publication.”

now reads:

“The codes are available at https://datadryad.org/stash/dataset/doi:10.5061%2Fdryad.fqz612jpp.”

The original Article has been corrected.

