# Supplementary material for: Degree of anisogamy is unrelated to the intensity of sexual selection
Source: Sci Rep. 2021 Sep 30;11:19424. doi: 10.1038/s41598-021-98616-2 (PMC8484679; doi:10.1038/s41598-021-98616-2)
Supplement: Supplementary file 1 — Supplementary Information. [file 41598_2021_98616_MOESM1_ESM.docx]

# **Supplementary material**

# Degree of anisogamy is unrelated to the intensity of sexual selection

Judit Mokos^1^, István Scheuring^1,2^, András Liker^3,4^, Robert P. Freckleton^5^ and Tamás Székely^6,7,*^

^1^ MTA-ELTE Theoretical Biology and Evolutionary Ecology Research Group, Eötvös Loránd University, Budapest, Hungary

^2^ Institute of Evolution, Centre for Ecological Research, Eötvös Loránd University, Budapest, Hungary

^3^ MTA-PE Evolutionary Ecology Research Group, University of Pannonia, Veszprém, Hungary

^4^ Behavioral Ecology Research Group, Center for Natural Science, University of Pannonia, Veszprém, Hungary

^5^ Department of Animal and Plant Sciences, University of Sheffield, Sheffield, United Kingdom

^6^ Milner Centre for Evolution, Department of Biology and Biochemistry, University of Bath, Bath, United Kingdom

^7^ Department of Evolutionary Zoology and Human Biology, University of Debrecen, Debrecen, Hungary

Corresponding author: Tamás Székely, [T.Szekely@bath.ac.uk](mailto:T.Szekely@bath.ac.uk)

Used abbreviation:

- GIB - gametic investment bias
- GSB - gamete size bias
- SSD - sexual size dimorphism
- PC - parental care
- dI_lnCVR - effect size of opportunity of selection
- dIs_lnCVR - effect size of opportunity of sexual selection
- dbeta_g - effect size of Bateman gradient
- PGLS - phylogenetically controlled generalized least square

# Data collection

We aimed to find all the species in the literature that’s selection indices or any data to calculate the selection indices (see below) was published. A species was included in the database only if at least one of its selection indices was available in the literature. The life-history traits data of these species were then collected also from the literature.

All the variables were handled as continuous variables. This method was chosen because it is a common practice in phylogenetical analysis, as variables coded on ordinal scales usually provide consistent results with continuous data. Besides, PGLSs are robust methods and they do not seem to be sensitive to ordinal vs continuous trait distributions (Garamszegi & Mundry, 2014).

## Selection indices

As Janicke et al. (Janicke et al., 2016) recently collected data of selection indices, we built on their species list of 66 species. To extend it, the Preferred Reporting Items for Systematic Reviews and Meta-Analyses (PRISMA) statement (Moher et al., 2009) was followed (for the PRISMA diagram see supplementary material Figure S1) using the same method given by Janicke et al. (2016) (Janicke et al., 2016). The ISI Web of Knowledge (Web of Science Core Collection, from 1945 to 2017) was searched in 2017 3rd of March the following “topic” search terms and format: (“Bateman*” OR “opportunit*for selection” OR“opportunit*for sexual selection”OR“selection gradient*”), where the asterisk (*) represents any group of characters, including no character. 754 candidate publications were obtained for further investigation. These studies were compared to Janicke et al’s database, and all the items absent were checked. The species list of Janicke et al was extended only with one new species (*Lamprotornis superbus* where data was extracted from the supplementary material of). Hermaphrodite species (*Physa acuta, Biomphalaria glabrata*) were excluded. Overall, the dataset used in this study contains data of 64 species from 9 classes of 4 animal phyla (see Figure 2).

# PRISMA diagram


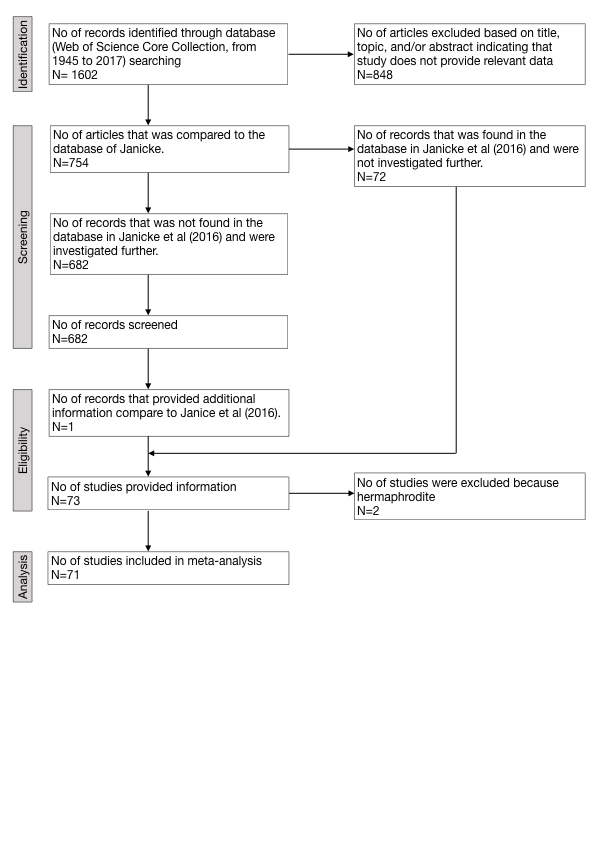


***Figure S1*** *PRISMA diagram showing the data collection.*

Following Janicke et al. (Janicke et al., 2016) three indices were used to indicate the intensity of selection: *opportunity for selection (I)* that is the standardized variance in reproductive success, *opportunity for sexual selection (I_s_)* that is the standardized variance in mating success and the *Bateman gradient (β_ss_)* that is the slope of an ordinary least-squares regression of reproductive success on mating success. For the statistical calculation, the effect sizes of these three indices (*ΔI, ΔIs*, and *Δβ_ss_*) were used. The effect size of these indices represents the bias of the intensity of selection between males and females, positive values indicate male bias. *ΔI* and *ΔI_s_* are the coefficient of variation ratio “lnCVR”, defined as the natural logarithm of the ratio between the coefficients of variation from males and females, and *Δβ_ss_* was calculated as *Hedges’ d (Hedges’ d* was computed as described in (Lajeunesse, 2013; Nakagawa et al., 2015))*.* Except for the species that was added by us (Lamprotornis superbus), all the selection indices used were taken from Janicke et al.

## Life-history traits

Data on life-history traits were collected using Web of Science. That included gamete size and body size of males and females, clutch size, testis size and parental care behaviour of males and females. Only data from published sources were used. The source of the data used is available in the published Dataset. In the case of species with missing data, values of close relative species were used (see Dataset), or if it was possible, the data was estimated based on other information (for example female gamete mass was calculated from diameter, or body mass was calculated using body length, see in Dataset).

### Gametic investment

Based on Liker et al (2015) (Liker et al., 2015), two types of indices were used to estimate gametic bias between the sexes: (i) the *gamete size bias index* which was calculated as log ([male gamete mass/male mass]/[female gamete mass/female mass]), whereas (ii) the *gametic investment bias* which was calculated as log([testis mass/male mass] / [female gamete mass * clutch size /female mass]). A negative value indicates female-biased gametic investment whereas the positive value a male-biased gametic investment.

To measure female gamete size, neonate weight or egg (fertilized ovum, including the weight of the shell and nutrition) weight were used, both in grams.

We understand that estimating male gametic investment is not straightforward, and sperm length and testes size may vary within individuals (Lüpold et al. 2016, Lüpold et al. 2020). Nevertheless, we aimed at using the best information that was available across a wide range of species and to estimate male gamete size, we used the volume of the sperm, (sperm length)^3^. Ideally, we were hoping to use sperm weight and ejaculate volumes, although as yet, these data are not available for the species in our analyses. The weight of the gamete is *c**(sperm length)^3^, where *c* is a constant depending on the geometry and the density of the gamete. If the length is measured in cm, then the density is about 1g/cm^3^, that is *c* is close to one in this sense. The length can be roughly at most ten times longer in one direction than to the other, so because of the geometry of the sperm *c* must be roughly between 0.01 to 1. To estimate the sensitivity of our results to the constant c, we have chosen *c*=0.1, 1, in a series of analysis. Probably because of logarithmic transformation in the indices we received no qualitative difference for different *c*-s, so the results are not sensitive to the choice of this parameter in a wide interval.

Clutch size (number of gametes per breeding event) and testis weight (in grams) were collected.

Please note that our dataset contains a wide range of species, both that shows female and male-biased gametic investment bias, also close to isogamous species, however close to isogamous species are underrepresented. For more details, please see the published Dataset.

The gamete size bias in our imputed dataset ranges from -23.32 to 6.61, whereas the gametic investment bias ranges from -11.23 to 6.92. Note that the majority of the species are anisogamous (see the Dataset).

### Sexual dimorphism

Sexual size dimorphism was calculated as log[(male size) / (female size)], thus positive value indicates male-biased species. Either the length or the mass of the body of males and females was collected. For calculating sexual size dimorphism, the size in the same dimension was used (for example male body length and female body length, or male body weight and female body weight). To compare the sexual size dimorphism index based on the length and based on the weight is valid as the weight = length^3^ * *c*, where *c* is a constant which is the same or very similar in both sexes.

### Parental care

A six-point-scale was used to estimate parental care bias based on Liker et al 2015 (Liker et al., 2015): 0 – female-only care, 1 – 1-33% male care, 2 – 34-66% male care, 3 – 77-99% male care, 4 –male-only care, NO – no parental care. The scores were based either on quantitative data where these were available or on qualitative description of parental care – see the justification in (Liker et al., 2015). If the statistical calculation allowed it, parental care data was treated as a factor variable (n=64). If not, then the species which does not provide parental care was not used in the statistical calculation and the variable was treated as interval scaled variables (n=37). This method was chosen because the *no caring* is orderable category compare to the species what provide parental care. However, in no care species both of the parents equally do not provide care, we decided not to merge *no care* species with nonbiased care species as the original status of the species is not known, and caring might be not equally shared in the evolutionary background, so lack of caring could be “non equally”.

## Phylogeny tree

To represent the phylogenetic relationships between species, the most recent comprehensive phylogeny was used from timetree.org (Hedges et al., 2006) that included all but seven species in the recent dataset. Table 1 contains the details of the added species.

***Table S1*** *Species added to the phylogeny trees.*

| **Added species** | **Source of phylogeny** |
| --- | --- |
| *Colpula lativentris* | (Li et al., 2012) |
| *Gerris gilettei* | (Li et al., 2012) |
| *Hippocampus subelongatus* | (Teske & Beheregaray, 2009) |
| *Ischnura gemina* | (Swaegers et al., 2014) |
| *Labidomera clivicollis* | (Kergoat et al., 2011) |
| *Megabruchidius dorsalis* | (Kergoat et al., 2011) |
| *Strongylocentrotus purpuratus* | (Lee, 2003) |

# Data imputation

As the main result of this study is a lack of pattern one could argue it is caused by the lack of data. A potential way to verify whether missing data is responsible for the lack of relationship is to fill the lack using imputation and comparing the result of the original and the completed dataset. If none of them shows the relationship it could be assumed the lack of relationship is not caused by the lack of data. Missing data also could result in low statistical power especially because PGLS discards any species with missing values reducing the sample size.

Imputation is a method when missing data is replaced with estimated data. It performs betters if the ratio of the missing data is low (Penone et al., 2014). To increase the reliability and accuracy of the imputation data of additional species were collected. These data were used only in the imputation but not in the later calculations. For the number of additional species, and the missing data ratio please see Table S2. For the list of the additional species please see the electronic supplementary materials.

Multiple imputation was performed based on the method of Jetz and Freckleton (2015) (Jetz & Freckleton, 2015), using the Brownian motion model and the lambda model. The results given by the two models were compared and no remarkable difference was found. For the used phylogeny tree please see the electronic supplementary materials. The missing values were drawn ten times from normal distribution using the *rnorm* function in R, resulting in ten completed datasets. Each of the datasets was analysed, and the mean and standard deviation of statistical indices, such as p-value, AIC, etc. were calculated. For the implementation of the imputation, the *Rphylopars* R package was used.

The results of the PGLSs using the original dataset and the imputed dataset using lambda model is reported in the paper. The results of the PGLSs using the imputed dataset using the Brownian motion model is reported in the supplementary material, below.

A leave-one-out cross-validation reliability check was performed to test the accuracy of the used imputation method. For this, the non-missing data points were deleted one by one and were imputed. The correlation between the original and imputed data points was investigated. The results of the correlation are shown in Table S2. According to this leave-one-out reliability check, the imputation is reliable as the correlation coefficients are higher than 0.8. For the R script of the leave-one-out cross-validation reliability check please see the electronic supplementary material.

***Table S2*** *The ratio of the imputed data, the number of the species used for imputation, and the result of the reliability check. To increase the accuracy of the imputation additional species’ data were used. For the reliability check, the original data points were left out and imputed, and the correlation between the original and imputed data points was tested. The correlation coefficients are reported in the table. Imputation was not necessary for parental care data.*

| Variable | The ratio of imputed data of the original 64 species | Number of species used in the imputation (including the original 64 species) | Correlation coefficients |
| --- | --- | --- | --- |
| Female body size | 0.312 | 5014 | 0.97 |
| Male body size | 0.203 | 4584 | 0.97 |
| Testis mass | 0.484 | 259 | 0.91 |
| Total sperm length | 0.359 | 489 | 0.82 |
| Clutch size | 0.266 | 12042 | 0.92 |
| Female gamete mass | 0.312 | 3966 | 0.97 |
| dI_lnCVR | 0.141 | Original 64 | - |
| dIs_lnCVR | 0.062 | Original 64 | - |
| dbeta_g | 0.234 | Original 64 | - |
| Parental care | 0 | Imputation was not used | - |

# Results using imputation using Brownian motion model

***Table S3*** *Phylogenetically corrected relationships between the elements of Darwin-Bateman paradigm. The multiple imputation using the Brownian motion model was used to generate ten full datasets and Phylogenetic Generalized Least Squares (PGLS) were performed on each dataset. The mean and standard deviation of the statistical indices were calculated. Parental care data were treated as interval scaled variable. If parental care data were used the species those do not show caring behaviour were excluded, then the sample size was 37. Otherwise, all 64 species were used. ΔI: opportunity of selection, ΔIs: opportunity for sexual selection, Δβ_ss_: Bateman gradient. The only trait that is associated with selection is parental care regardless of which indices were used.*

| **formula** | **explanatory variable** | **estimate** | **std.error** | **t** | **p** | **R^2^** | **adjusted R^2^** | **𝜆** | **F** | **AIC** | **AICc** |
| --- | --- | --- | --- | --- | --- | --- | --- | --- | --- | --- | --- |
| dI_lnCVR ~ GSB + SSD + PC | Intercept | 0.78 ± 0.17 | 0.25 ± 0.08 | 3.48 ± 1.31 | 0.04* ± 0.10 | 0.41 ± 0.10 | 0.36 ± 0.11 | 0.08 ± 0.26 | 8.07 ± 2.93 | 47.83 | 49.08 |
|  | GI | 0.00 ± 0.01 | 0.01 ± 0.00 | 0.27 ± 0.59 | 0.59 ± 0.20 |  |  |  |  |  |  |
|  | SSD | -0.09 ± 0.12 | 0.11 ± 0.06 | -0.57 ± 0.77 | 0.52 ± 0.29 |  |  |  |  |  |  |
|  | PC | -0.25 ± 0.04 | 0.06 ± 0.01 | -4.51 ± 1.07 | 0.00* ± 0.01 |  |  |  |  |  |  |
| dI_lnCVR ~ GIB + SSD + PC | Intercept | 0.75 ± 0.13 | 0.16 ± 0.09 | 5.58 ± 1.84 | 0.03* ± 0.10 | 0.41 ± 0.10 | 0.36 ± 0.11 | 0.08 ± 0.24 | 8.22 ± 3.06 | 46.67 | 47.92 |
|  | GI | 0.00 ± 0.01 | 0.02 ± 0.00 | 0.04 ± 0.81 | 0.56 ± 0.28 |  |  |  |  |  |  |
|  | SSD | -0.11 ± 0.12 | 0.11 ± 0.06 | -0.74 ± 0.94 | 0.40 ± 0.29 |  |  |  |  |  |  |
|  | PC | -0.25 ± 0.04 | 0.06 ± 0.01 | -4.48 ± 1.06 | 0.00* ± 0.01 |  |  |  |  |  |  |
| dIs_lnCVR ~ GSB + SSD + PC | Intercept | 0.48 ± 0.14 | 0.21 ± 0.03 | 2.33 ± 0.67 | 0.06 ± 0.09 | 0.33 ± 0.01 | 0.26 ± 0.02 | 0.00 ± 0.00 | 5.31 ± 0.36 | 55.36 | 56.61 |
|  | GI | -0.01 ± 0.01 | 0.01 ± 0.00 | -0.51 ± 0.67 | 0.52 ± 0.27 |  |  |  |  |  |  |
|  | SSD | 0.01 ± 0.09 | 0.11 ± 0.06 | 0.10 ± 0.97 | 0.45 ± 0.25 |  |  |  |  |  |  |
|  | PC | -0.20 ± 0.01 | 0.05 ± 0.00 | -3.71 ± 0.20 | 0.00* ± 0.00 |  |  |  |  |  |  |
| dIs_lnCVR ~ GIB + SSD + PC | Intercept | 0.63 ± 0.05 | 0.12 ± 0.01 | 5.04 ± 0.48 | 0.00* ± 0.00 | 0.34 ± 0.03 | 0.28 ± 0.03 | 0.00 ± 0.00 | 5.60 ± 0.65 | 55.53 | 56.78 |
|  | GI | 0.01 ± 0.01 | 0.02 ± 0.00 | 0.85 ± 0.77 | 0.42 ± 0.31 |  |  |  |  |  |  |
|  | SSD | 0.05 ± 0.09 | 0.10 ± 0.06 | 0.40 ± 0.95 | 0.46 ± 0.28 |  |  |  |  |  |  |
|  | PC | -0.20 ± 0.01 | 0.05 ± 0.00 | -3.76 ± 0.17 | 0.00* ± 0.00 |  |  |  |  |  |  |
| dbeta_g ~ GSB + SSD + PC | Intercept | 0.78 ± 0.24 | 0.26 ± 0.08 | 3.10 ± 0.95 | 0.04* ± 0.09 | 0.39 ± 0.11 | 0.33 ± 0.12 | 0.24 ± 0.26 | 7.66 ± 3.92 | 59.85 | 61.10 |
|  | GI | 0.01 ± 0.01 | 0.01 ± 0.00 | 0.63 ± 0.98 | 0.45 ± 0.36 |  |  |  |  |  |  |
|  | SSD | 0.07 ± 0.25 | 0.11 ± 0.07 | 0.30 ± 2.27 | 0.29 ± 0.37 |  |  |  |  |  |  |
|  | PC | -0.22 ± 0.04 | 0.06 ± 0.01 | -3.96 ± 0.75 | 0.00* ± 0.00 |  |  |  |  |  |  |
| dbeta_g ~ GIB + SSD + PC | Intercept | 0.67 ± 0.15 | 0.20 ± 0.11 | 4.36 ± 2.47 | 0.05 ± 0.12 | 0.39 ± 0.12 | 0.33 ± 0.14 | 0.22 ± 0.29 | 7.87 ± 4.86 | 58.55 | 59.80 |
|  | GI | 0.00 ± 0.02 | 0.02 ± 0.01 | 0.41 ± 1.20 | 0.45 ± 0.34 |  |  |  |  |  |  |
|  | SSD | 0.04 ± 0.25 | 0.11 ± 0.07 | 0.24 ± 2.42 | 0.27 ± 0.31 |  |  |  |  |  |  |
|  | PC | -0.22 ± 0.04 | 0.06 ± 0.01 | -3.92 ± 0.84 | 0.00* ± 0.00 |  |  |  |  |  |  |

| **formula** | **explanatory variable** | **estimate** | **std.error** | **t** | **p** | **R^2^** | **adjusted R^2^** | **𝜆** | **F** | **AIC** | **AICc** |
| --- | --- | --- | --- | --- | --- | --- | --- | --- | --- | --- | --- |
| dI_lnCVR~ GSB | intercept | 0.45 ± 0.03 | 0.17 ± 0.05 | 2.97 ± 1.29 | 0.03* ± 0.03 | 0.01 ± 0.01 | -0.01 ± 0.01 | 0.27 ± 0.18 | 0.31 ± 0.38 | 96.86 | 97.05 |
|  | variable | 0.00 ± 0.00 | 0.01 ± 0.00 | 0.03 ± 0.58 | 0.67 ± 0.23 |  |  |  |  |  |  |
| dI_lnCVR~ GIB | intercept | 0.46 ± 0.02 | 0.16 ± 0.05 | 3.30 ± 1.75 | 0.02* ± 0.01 | 0.01 ± 0.01 | -0.01 ± 0.01 | 0.26 ± 0.17 | 0.49 ± 0.49 | 96.32 | 96.51 |
|  | variable | 0.00 ± 0.01 | 0.01 ± 0.00 | 0.33 ± 0.65 | 0.58 ± 0.25 |  |  |  |  |  |  |
| dIs_lnCVR~ GSB | intercept | 0.25 ± 0.19 | 0.16 ± 0.11 | 1.88 ± 1.00 | 0.16 ± 0.26 | 0.04 ± 0.03 | 0.03 ± 0.03 | 0.21 ± 0.33 | 2.70 ± 2.30 | 89.12 | 89.32 |
|  | variable | -0.01 ± 0.01 | 0.01 ± 0.00 | -1.03 ± 1.34 | 0.25 ± 0.26 |  |  |  |  |  |  |
| dIs_lnCVR~ GIB | intercept | 0.31 ± 0.17 | 0.14 ± 0.13 | 3.27 ± 1.64 | 0.11 ± 0.29 | 0.05 ± 0.06 | 0.03 ± 0.06 | 0.21 ± 0.34 | 3.42 ± 4.98 | 92.13 | 92.32 |
|  | variable | 0.00 ± 0.01 | 0.01 ± 0.00 | 0.42 ± 1.90 | 0.29 ± 0.30 |  |  |  |  |  |  |
| dbeta_g~ GSB | intercept | 0.42 ± 0.09 | 0.10 ± 0.01 | 4.24 ± 0.93 | 0.00* ± 0.00 | 0.01 ± 0.01 | -0.01 ± 0.01 | 0.01 ± 0.02 | 0.55 ± 0.56 | 107.55 | 107.74 |
|  | variable | 0.00 ± 0.01 | 0.01 ± 0.00 | 0.23 ± 0.74 | 0.54 ± 0.22 |  |  |  |  |  |  |
| dbeta_g~ GIB | intercept | 0.42 ± 0.06 | 0.09 ± 0.03 | 4.86 ± 1.08 | 0.00* ± 0.00 | 0.01 ± 0.02 | -0.01 ± 0.02 | 0.03 ± 0.06 | 0.63 ± 1.23 | 107.97 | 108.17 |
|  | variable | 0.00 ± 0.01 | 0.01 ± 0.00 | 0.37 ± 0.74 | 0.61 ± 0.27 |  |  |  |  |  |  |

| **formula** | **explanatory variable** | **estimate** | **std.error** | **t** | **p** | **R^2^** | **adjusted R^2^** | **𝜆** | **F** | **AIC** | **AICc** |
| --- | --- | --- | --- | --- | --- | --- | --- | --- | --- | --- | --- |
| PC~ dI_lnCVR | Intercept | 1.84 ± 0.19 | 1.48 ± 0.10 | 1.25 ± 0.19 | 0.23 ± 0.07 | 0.27 ± 0.07 | 0.25 ± 0.07 | 0.99 ± 0.01 | 13.16 ± 4.51 | 114.84 | 115.19 |
|  | Variable | -0.96 ± 0.15 | 0.27 ± 0.02 | -3.58 ± 0.65 | 0.00* ± 0.01 |  |  |  |  |  |  |
| PC~ dIs_lnCVR | Intercept | 1.59 ± 0.01 | 1.43 ± 0.04 | 1.12 ± 0.03 | 0.27 ± 0.01 | 0.19 ± 0.01 | 0.17 ± 0.01 | 0.97 ± 0.01 | 8.14 ± 0.79 | 121.00 | 121.35 |
|  | Variable | -0.89 ± 0.02 | 0.31 ± 0.01 | -2.85 ± 0.14 | 0.01* ± 0.00 |  |  |  |  |  |  |
| PC~ dbeta_g | Intercept | 1.62 ± 0.34 | 1.43 ± 0.15 | 1.16 ± 0.35 | 0.28 ± 0.11 | 0.28 ± 0.09 | 0.25 ± 0.09 | 0.98 ± 0.02 | 13.94 ± 6.04 | 120.50 | 120.85 |
|  | Variable | -1.00 ± 0.15 | 0.28 ± 0.04 | -3.65 ± 0.82 | 0.00 *± 0.01 |  |  |  |  |  |  |
| SSD~ dI_lnCVR | Intercept | -0.52 ± 1.42 | 0.83 ± 0.56 | -0.88 ± 1.55 | 0.36 ± 0.37 | 0.00 ± 0.01 | -0.03 ± 0.01 | 0.99 ± 0.00 | 0.13 ± 0.26 | 108.91 | 109.26 |
|  | Variable | -0.01 ± 0.07 | 0.15 ± 0.10 | -0.12 ± 0.36 | 0.81 ± 0.18 |  |  |  |  |  |  |
| SSD~ dIs_lnCVR | Intercept | -0.52 ± 1.44 | 0.83 ± 0.56 | -0.89 ± 1.56 | 0.36 ± 0.37 | 0.00 ± 0.00 | -0.03 ± 0.00 | 0.99 ± 0.00 | 0.02 ± 0.02 | 108.92 | 109.28 |
|  | Variable | 0.01 ± 0.02 | 0.16 ± 0.11 | 0.06 ± 0.12 | 0.91 ± 0.06 |  |  |  |  |  |  |
| SSD~ dbeta_g | Intercept | -0.50 ± 1.41 | 0.83 ± 0.56 | -0.90 ± 1.54 | 0.35 ± 0.35 | 0.02 ± 0.02 | -0.01 ± 0.02 | 0.99 ± 0.00 | 0.60 ± 0.64 | 108.12 | 108.48 |
|  | Variable | 0.01 ± 0.13 | 0.15 ± 0.10 | 0.23 ± 0.78 | 0.54 ± 0.25 |  |  |  |  |  |  |

# Descriptive statistics


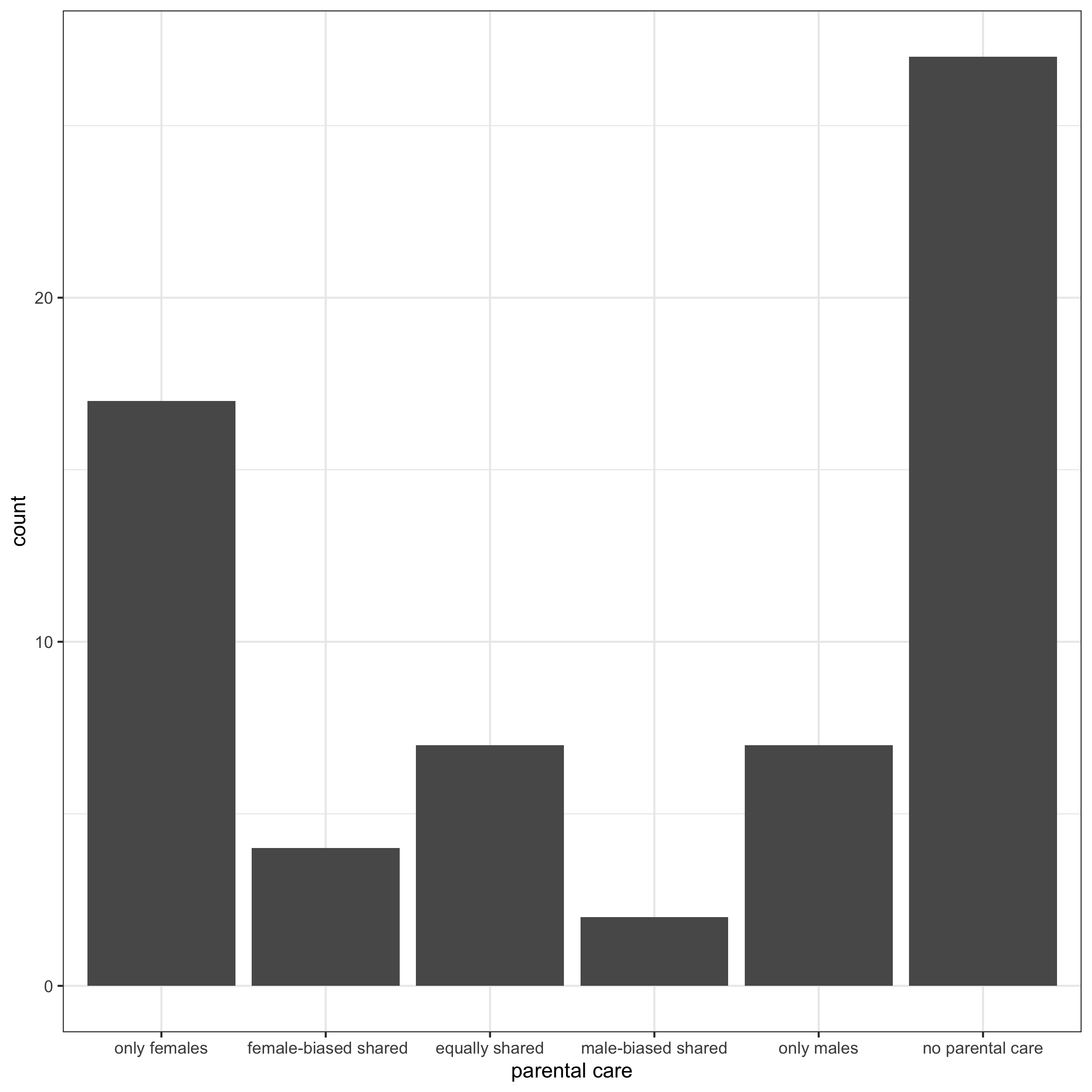


***Figure S2*** *Histograms of parental care.*


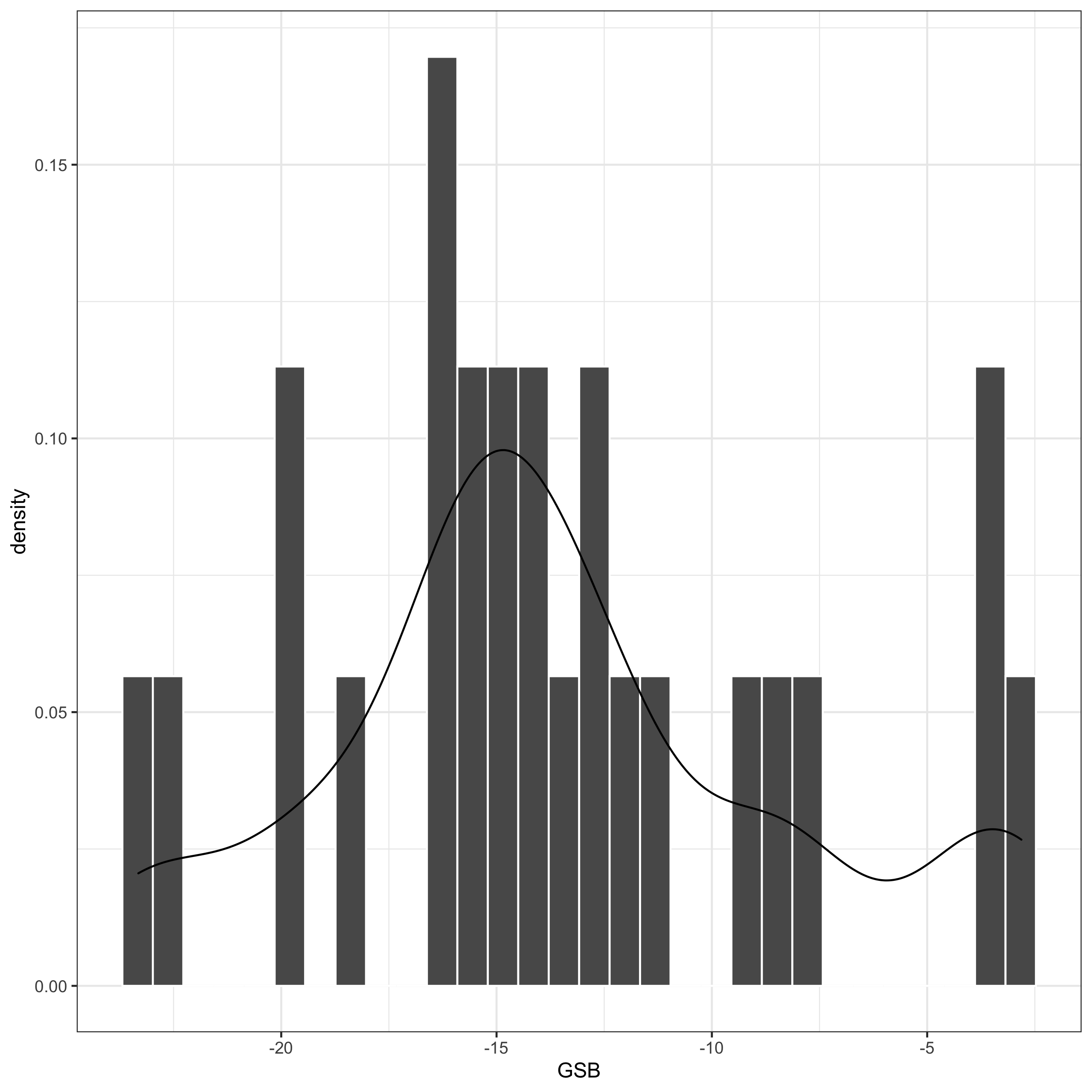


***Figure S3*** *Histograms of game size bias.*


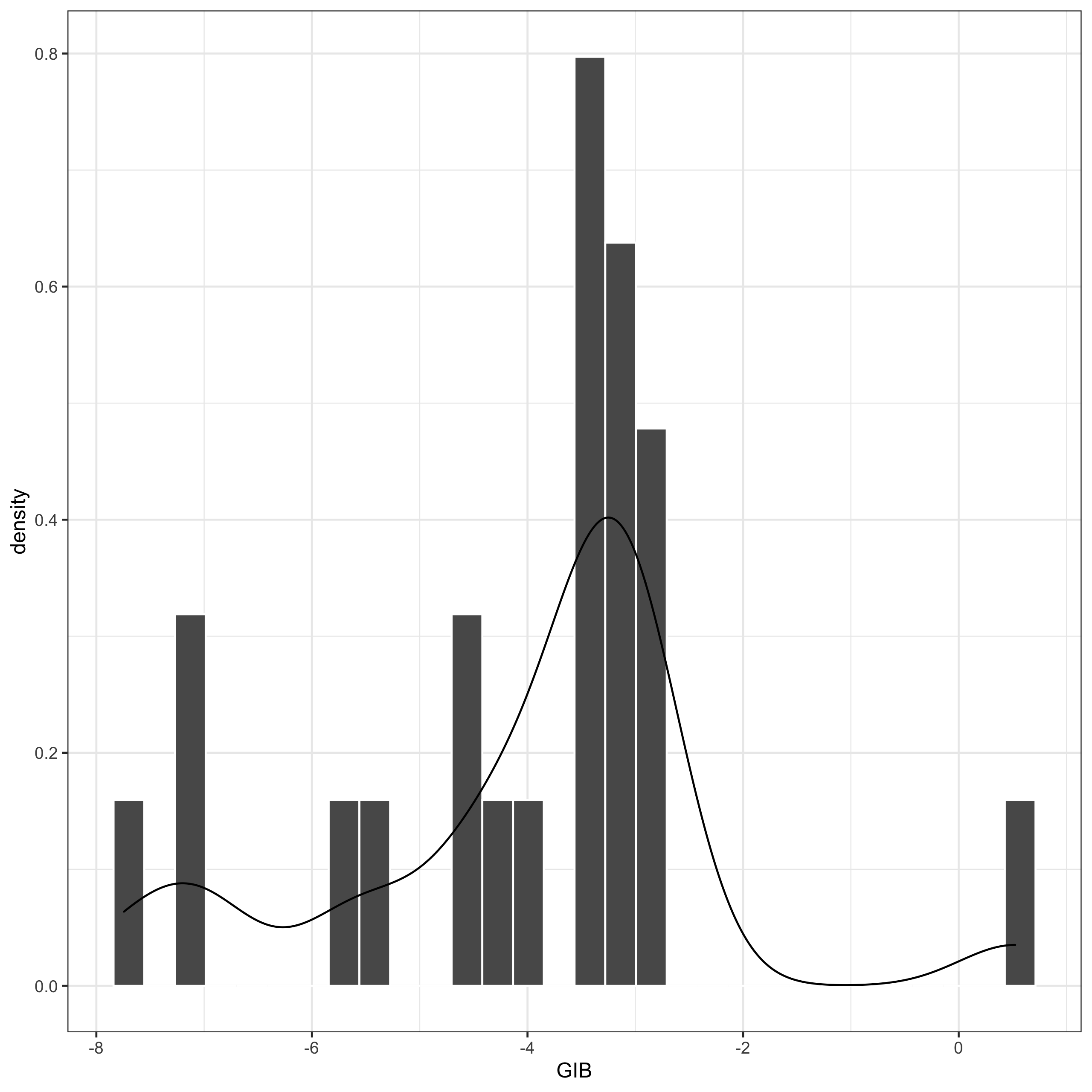


***Figure S4*** *Histograms of gametic investment bias.*


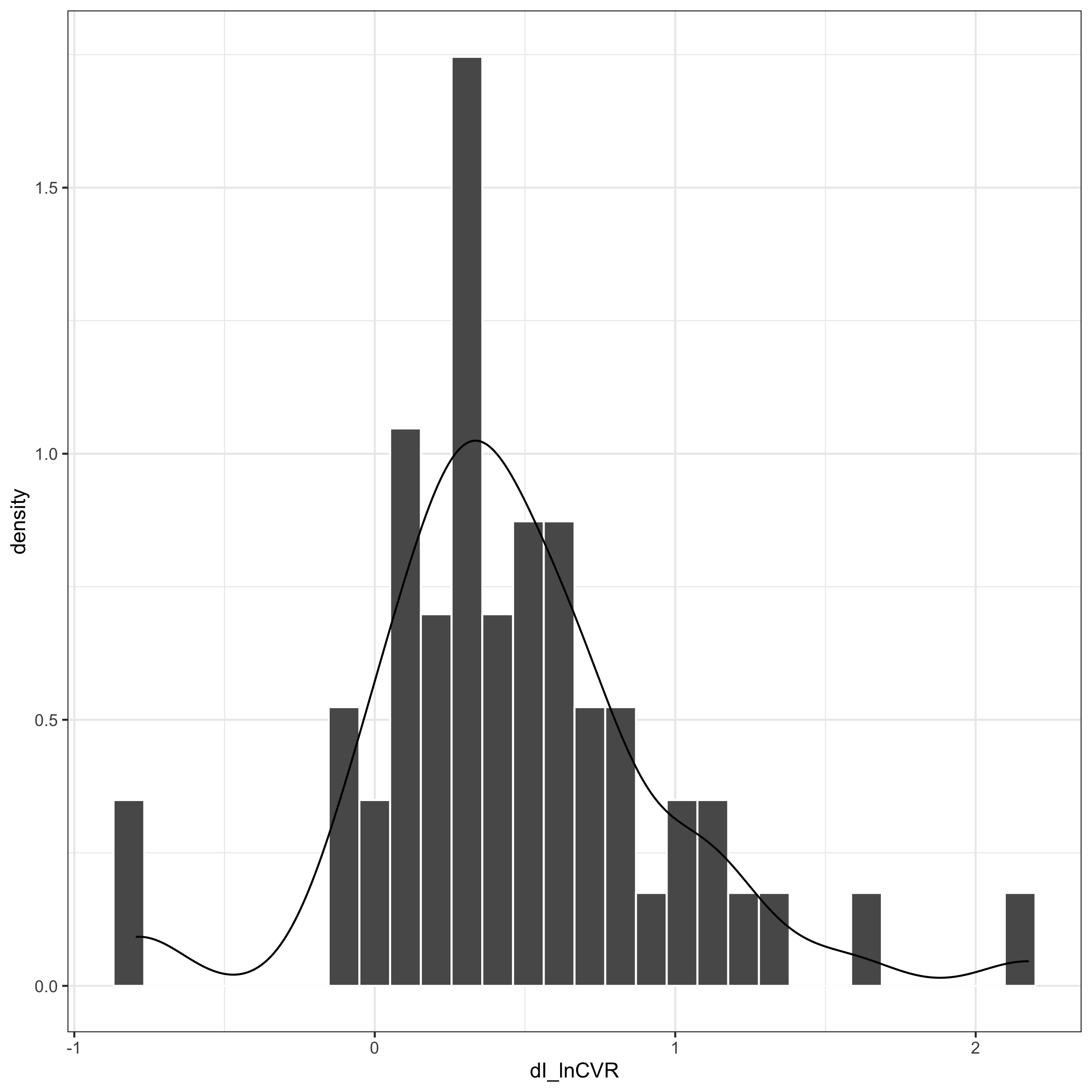


***Figure S5*** *Histograms of sexual selection index.*


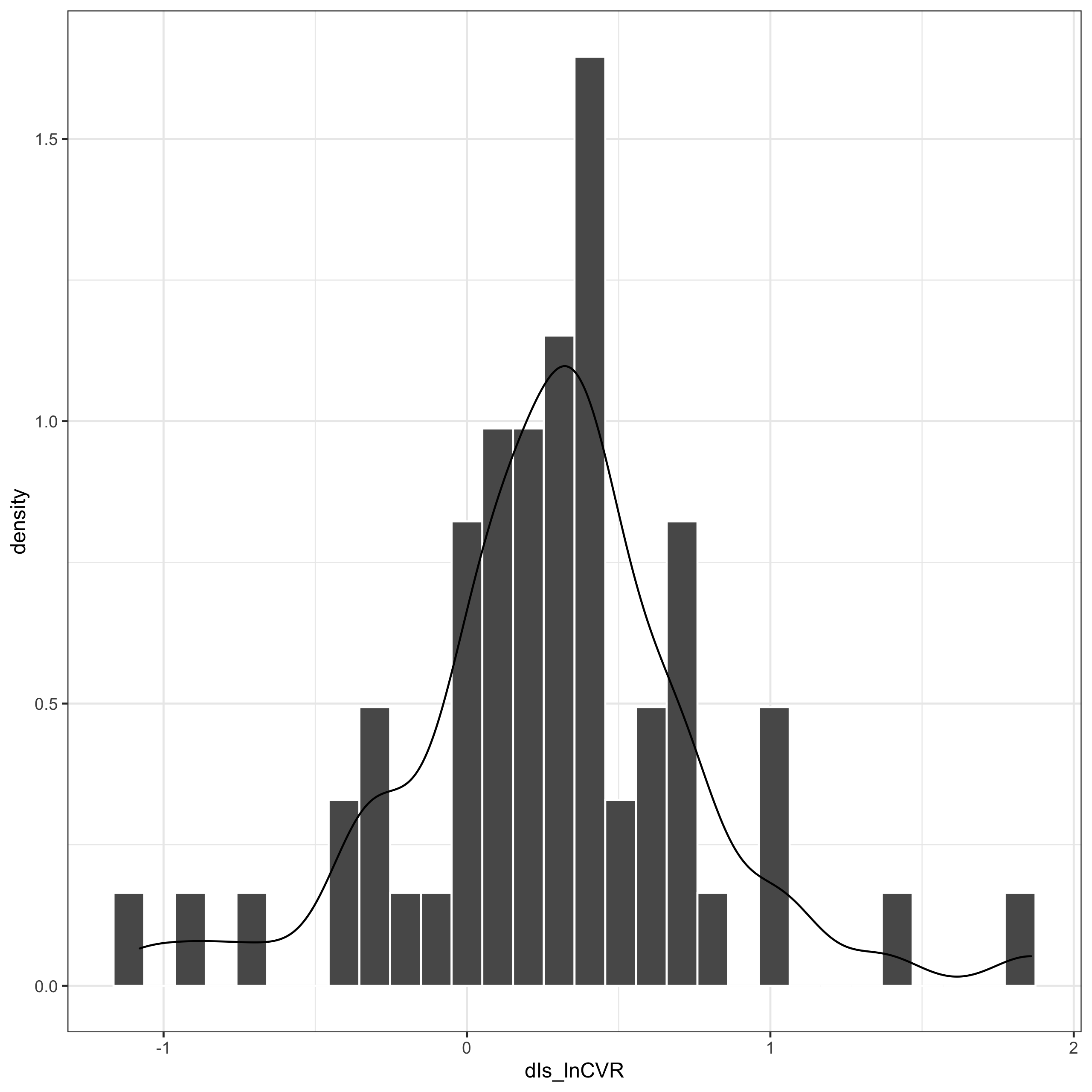


***Figure S6*** *Histograms of sexual selection index.*


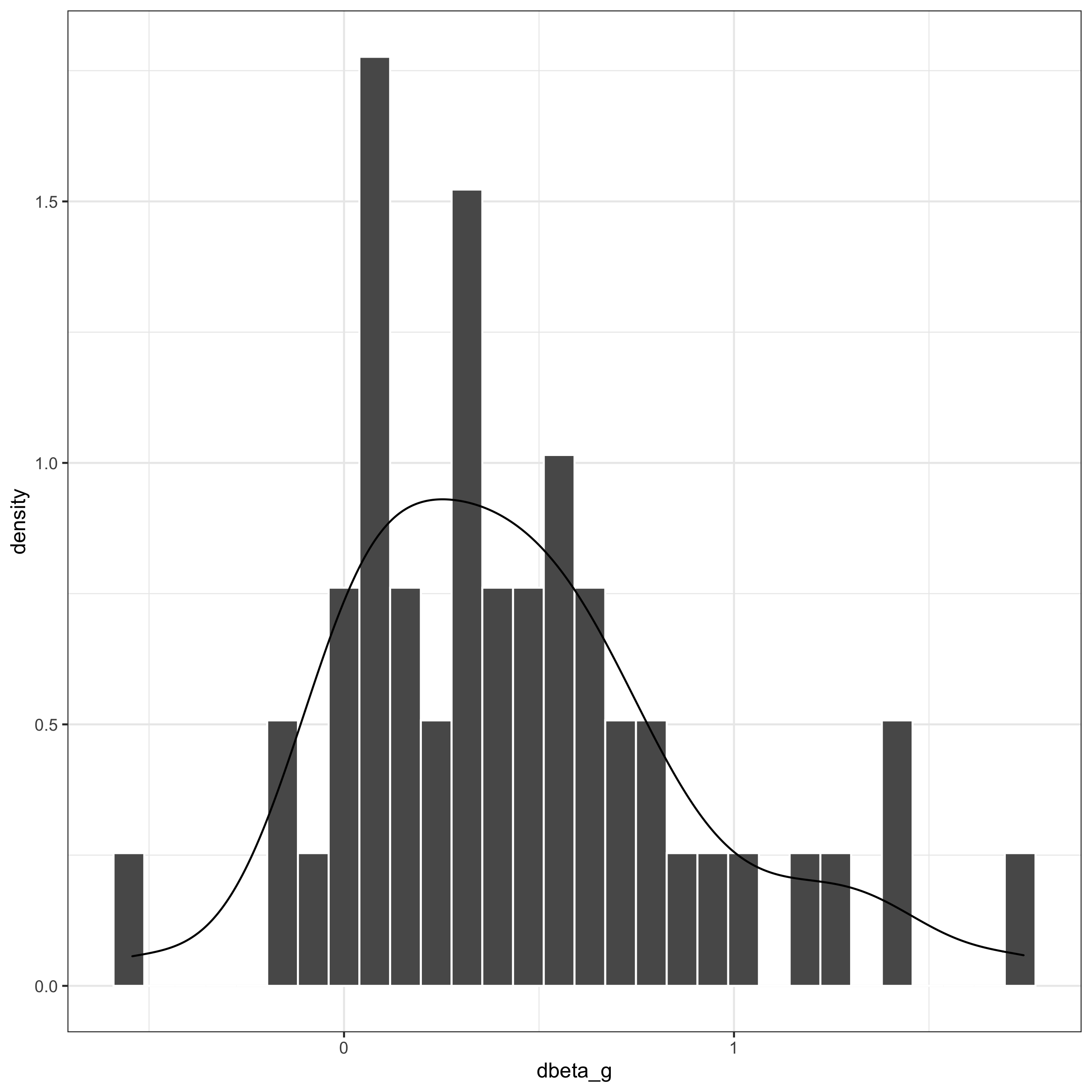


***Figure S7*** *Histograms of Bateman gradient.*

***Table S4*** *Correlation coefficients between variables. Spearman correlation was used.*

| **rownames** | **GIB** | **GSB** | **SSD** | **dI_lnCVR** | **dIs_lnCVR** | **dbeta_g** |
| --- | --- | --- | --- | --- | --- | --- |
| **GIB** | 1.00 | -0.09 | -0.02 | 0.13 | -0.04 | 0.10 |
| **GSB** | -0.09 | 1.00 | -0.43 | -0.04 | -0.34 | -0.03 |
| **SSD** | -0.02 | -0.43 | 1.00 | -0.00 | 0.19 | 0.15 |
| **dI_lnCVR** | 0.13 | -0.04 | -0.00 | 1.00 | 0.55 | 0.83 |
| **dIs_lnCVR** | -0.04 | -0.34 | 0.19 | 0.55 | 1.00 | 0.53 |
| **dbeta_g** | 0.10 | -0.03 | 0.15 | 0.83 | 0.53 | 1.00 |


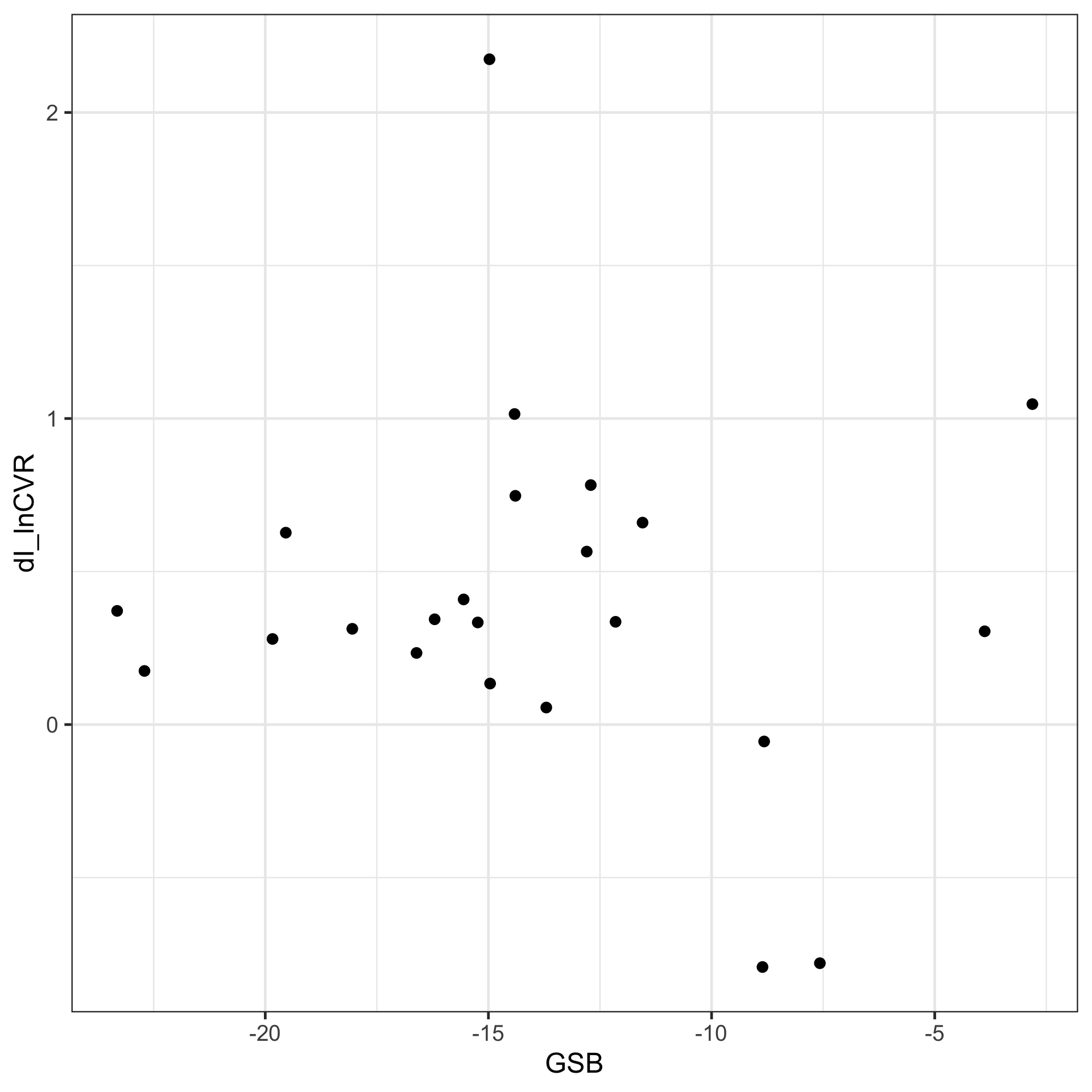


***Figure S8*** *Relationship between sexual selection index and anisogamy index.*


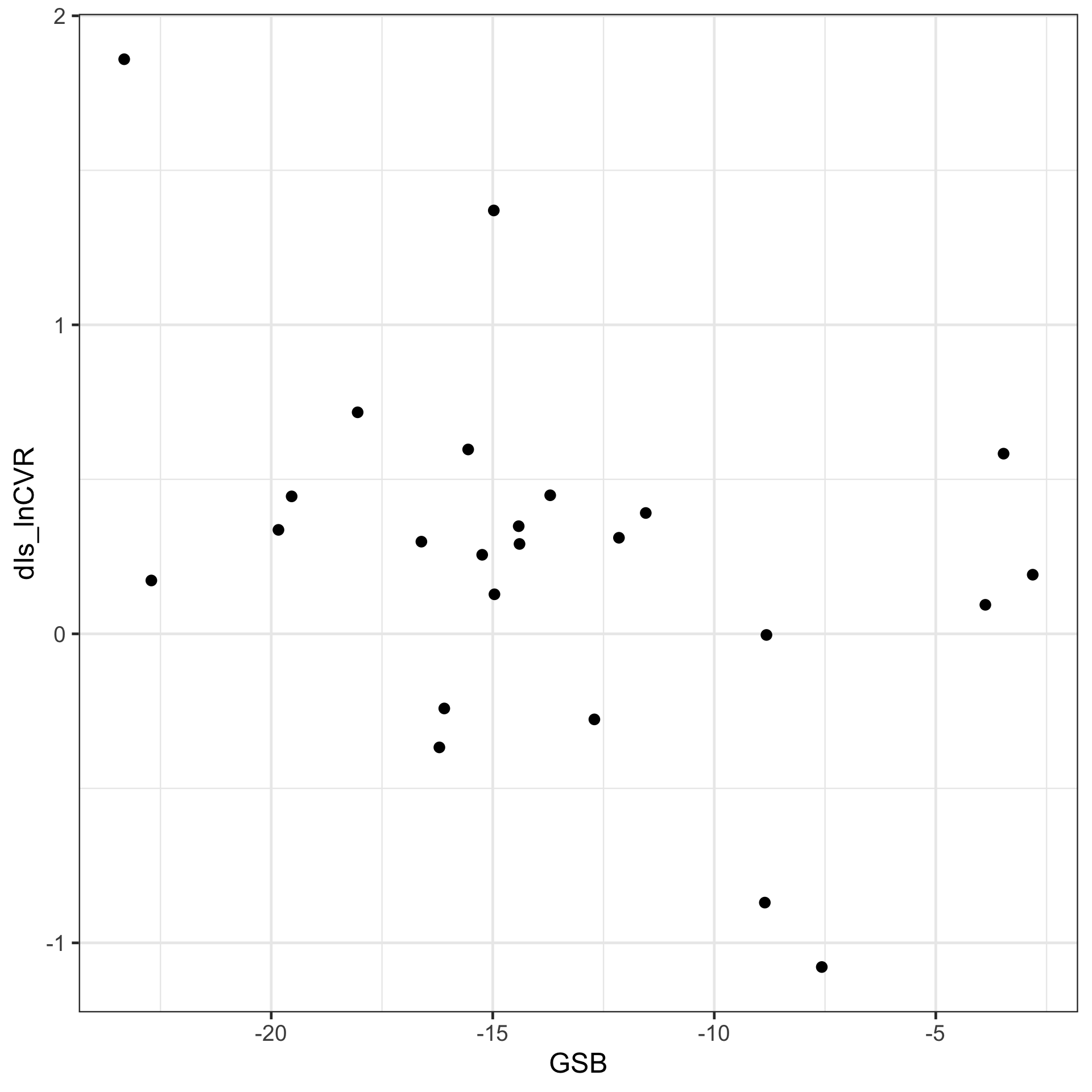


***Figure S9*** *Relationship between sexual selection index and anisogamy index.*


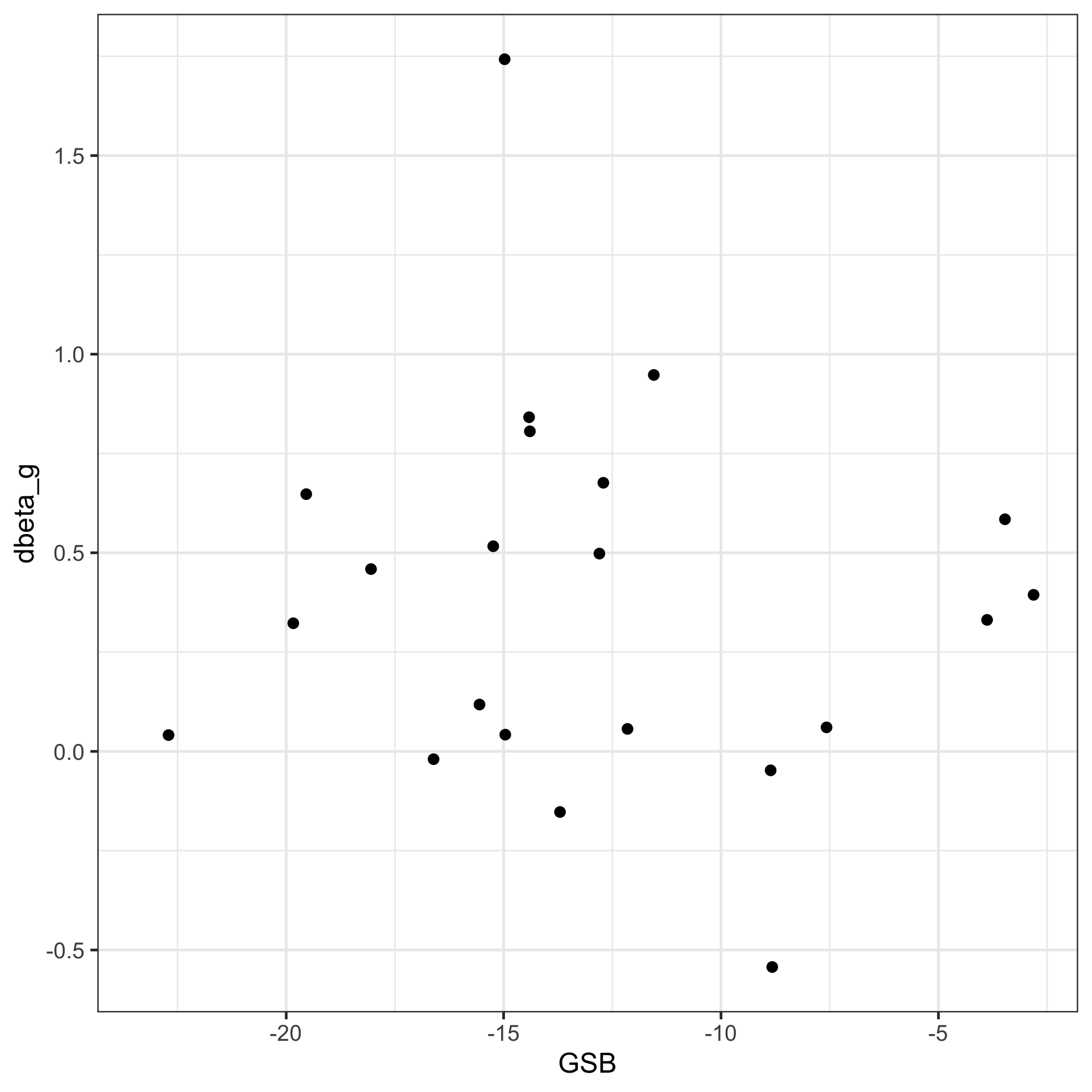


***Figure S10*** *Relationship between sexual selection index and anisogamy index.*


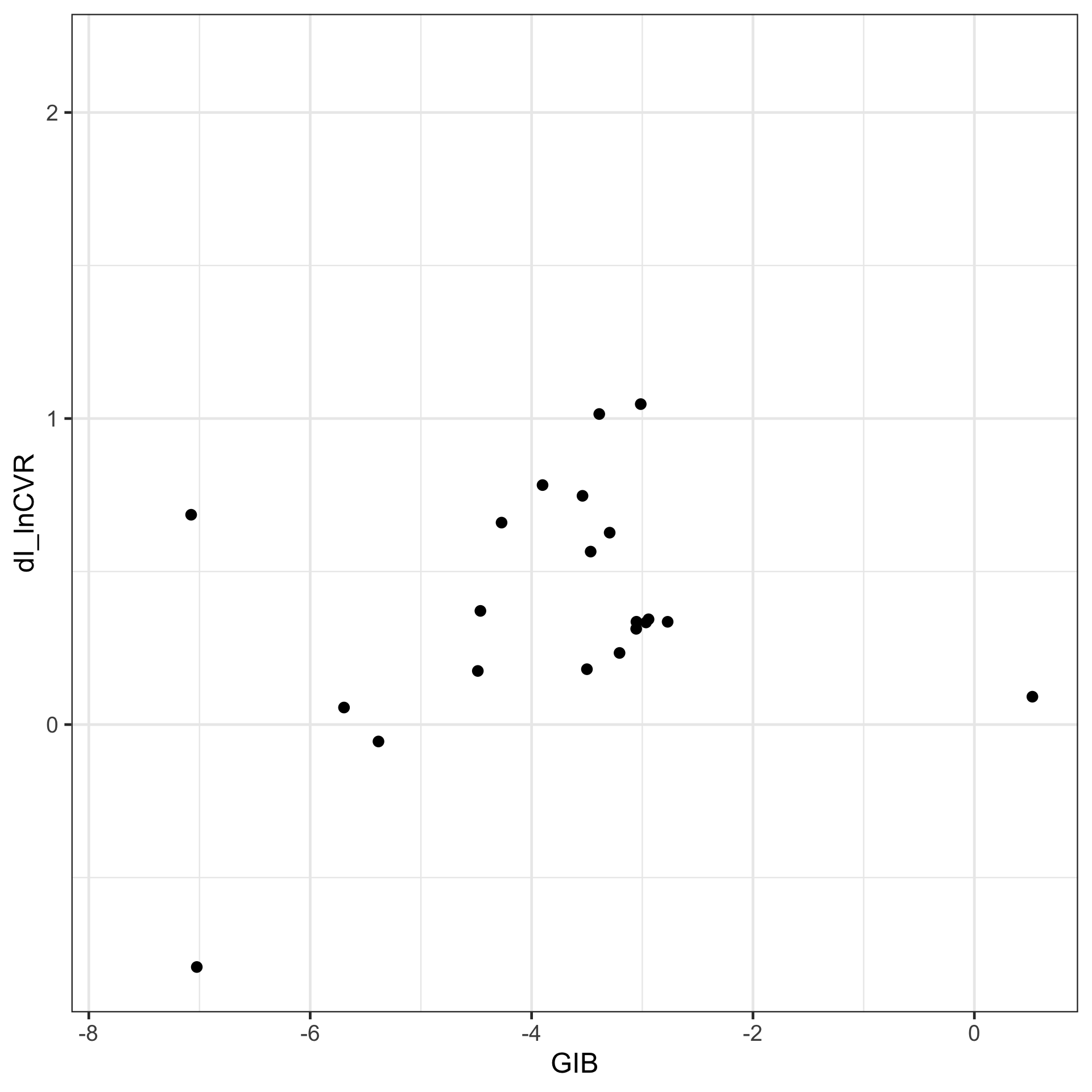


***Figure S11*** *Relationship between sexual selection index and anisogamy index.*


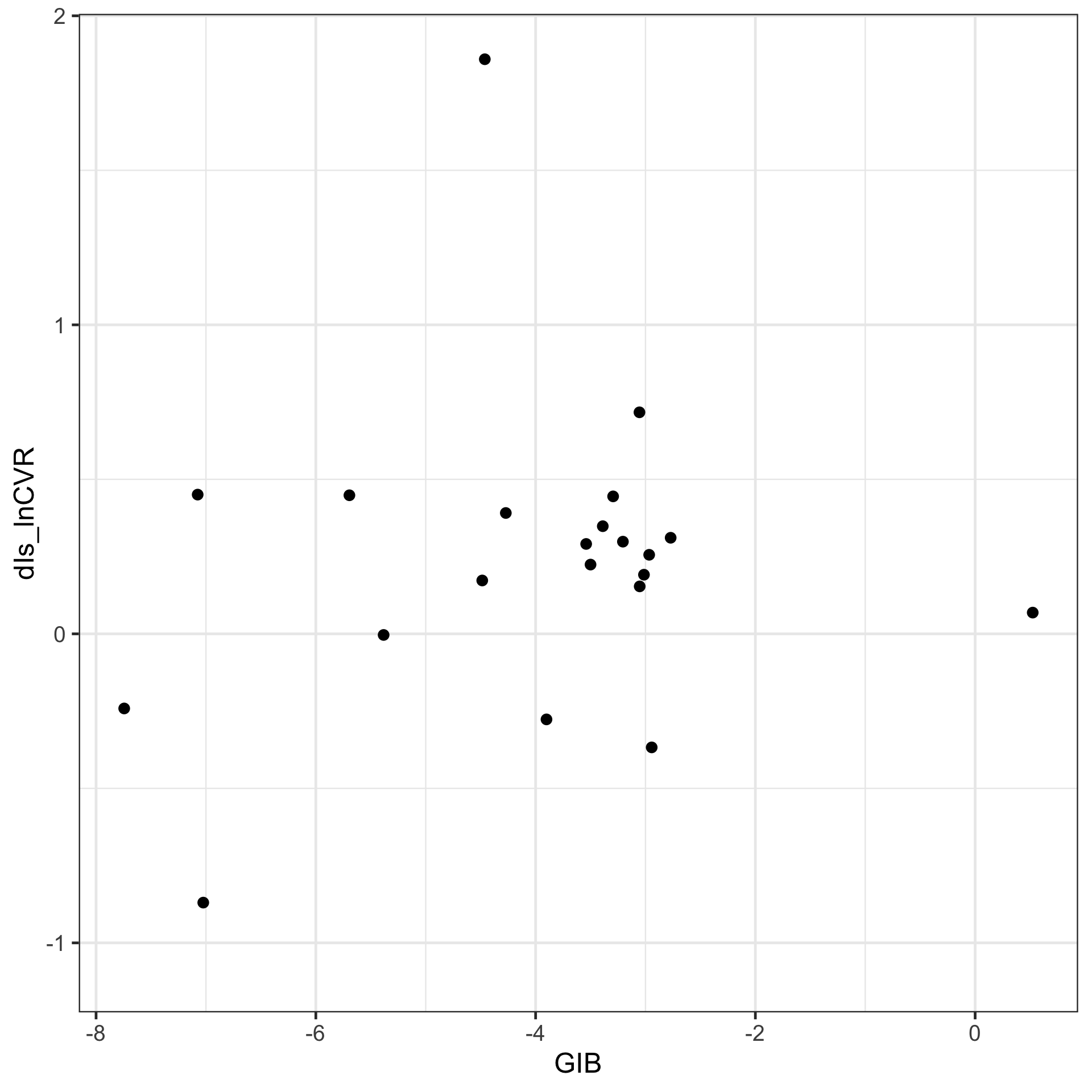


***Figure S12*** *Relationship between sexual selection index and anisogamy index.*


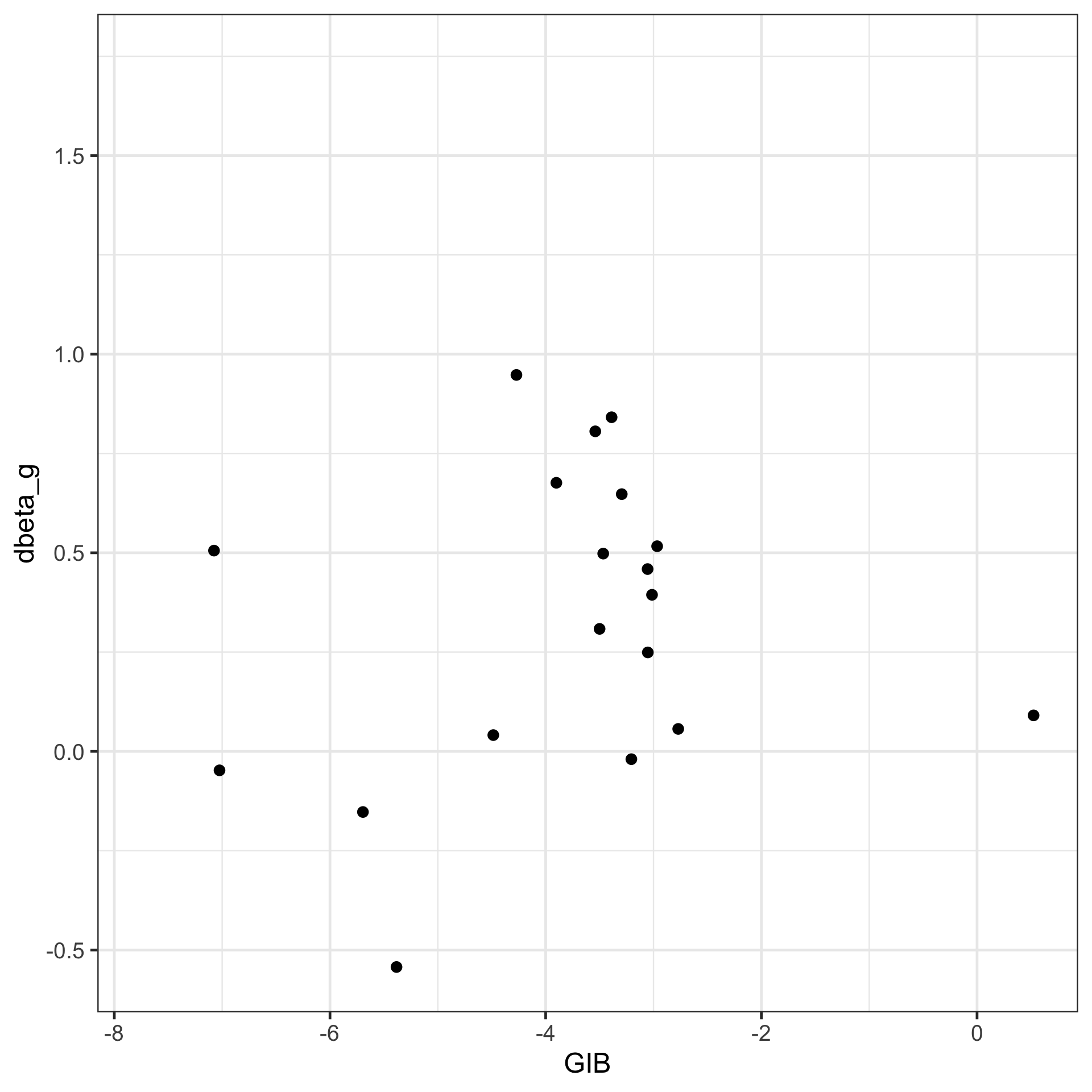


***Figure S13*** *Relationship between sexual selection index and anisogamy index.*

# References

Apakupakul, K., & Rubenstein, D. R. (2015). Bateman’s principle is reversed in a cooperatively breeding bird. *Biology Letters*, *11*(4), 20150034. https://doi.org/10.1098/rsbl.2015.0034

Garamszegi, L. Z., & Mundry, R. (2014). Multimodel-Inference in Comparative Analyses. In L. Z. Garamszegi (Ed.), *Modern Phylogenetic Comparative Methods and Their Application in Evolutionary Biology*. Springer Berlin Heidelberg. https://doi.org/10.1007/978-3-662-43550-2_12

Goolsby, E. W., Bruggeman, J., & Ané, C. (2017). Rphylopars: fast multivariate phylogenetic comparative methods for missing data and within-species variation. *Methods in Ecology and Evolution*, *8*(1), 22–27. https://doi.org/10.1111/2041-210X.12612

Hedges, S. B., Dudley, J., & Kumar, S. (2006). TimeTree: A public knowledge-base of divergence times among organisms. *Bioinformatics*, *22*(23), 2971–2972. https://doi.org/10.1093/bioinformatics/btl505

Janicke, T., Häderer, I. K., Lajeunesse, M. J., & Anthes, N. (2016). Darwinian sex roles confirmed across the animal kingdom. *Science Advances*, *2*(2), e1500983–e1500983. https://doi.org/10.1126/sciadv.1500983

Jetz, W., & Freckleton, R. P. (2015). Towards a general framework for predicting threat status of data-deficient species from phylogenetic, spatial and environmental information. *Philosophical Transactions of the Royal Society B: Biological Sciences*, *370*(1662), 20140016–20140016. https://doi.org/10.1098/rstb.2014.0016

Kergoat, G. J., Le Ru, B. P., Genson, G., Cruaud, C., Couloux, A., & Delobel, A. (2011). Phylogenetics, species boundaries and timing of resource tracking in a highly specialized group of seed beetles (Coleoptera: Chrysomelidae: Bruchinae). *Molecular Phylogenetics and Evolution*, *59*(3), 746–760. https://doi.org/10.1016/j.ympev.2011.03.014

Lajeunesse, M. (2013). Recovering missing data or partial data from studies: A survey of conversions and imputation for meta-analysis. *Handbook of Meta-Analysis in Ecology and Evolution*, 195–206.

Lee, Y. H. (2003). Molecular phylogenies and divergence times of sea urchin species of Strongylocentrotidae, Echinoida. *Molecular Biology and Evolution*, *20*(8), 1211–1221. https://doi.org/10.1093/molbev/msg125

Lüpold, S., de Boer, R.A., Evans, J.P., Tomkins, J.L. & Fitzpatrick, J.L. (2020) How sperm competition shapes the evolution of testes and sperm: a meta-analysis. *Philosophical Transactions of the Royal Society B: Biological Sciences* 375:20200064.

Lüpold, S., Manier, M.K., Puniamoorthy, N., Schoff, C., Starmer, W.T., Luepold, S.H.B., Belote, J.M., & Pitnick, S. (2016) How sexual selection can drive the evolution of costly sperm ornamentation. *Nature* 533:535-538

Li, M., Tian, Y., Zhao, Y., & Bu, W. (2012). Higher level phylogeny and the first divergence time estimation of heteroptera (insecta: Hemiptera) based on multiple genes. *PLoS ONE*, *7*(2). https://doi.org/10.1371/journal.pone.0032152

Liker, A., Freckleton, R. P., Remeš, V., & Székely, T. (2015). Sex differences in parental care: Gametic investment, sexual selection, and social environment. *Evolution*, *69*(11), 2862–2875. https://doi.org/10.1111/evo.12786

Moher, D., Liberati, A., Tetzlaff, J., Altman, D. G., Altman, D., Antes, G., … Tugwell, P. (2009). Preferred reporting items for systematic reviews and meta-analyses: The PRISMA statement. *PLoS Medicine*, *6*(7). https://doi.org/10.3736/jcim20090918

Nakagawa, S., Poulin, R., Mengersen, K., Reinhold, K., Engqvist, L., Lagisz, M., & Senior, A. M. (2015). Meta-analysis of variation: Ecological and evolutionary applications and beyond. *Methods in Ecology and Evolution*, *6*(2), 143–152. https://doi.org/10.1111/2041-210X.12309

Penone, C., Davidson, A. D., Shoemaker, K. T., Di Marco, M., Rondinini, C., Brooks, T. M., … Costa, G. C. (2014). Imputation of missing data in life-history trait datasets: Which approach performs the best? *Methods in Ecology and Evolution*, *5*(9), 1–10. https://doi.org/10.1111/2041-210X.12232

Swaegers, J., Janssens, S. B., Ferreira, S., Watts, P. C., Mergeay, J., McPeek, M. A., & Stoks, R. (2014). Ecological and evolutionary drivers of range size in Coenagrion damselflies. *Journal of Evolutionary Biology*, *27*(11), 2386–2395. https://doi.org/10.1111/jeb.12481

Teske, P. R., & Beheregaray, L. B. (2009). Evolution of seahorses’ upright posture was linked to Oligocene expansion of seagrass habitats. *Biology Letters*, *5*(4), 521–523. https://doi.org/10.1098/rsbl.2009.0152
